# Thermal Aging Behavior of PFA-Insulated Cables for Future Electrified Aircraft: From Material Evolution to Cable-Level Mechanical Response

**DOI:** 10.3390/polym18192382

**Published:** 2026-09-29

**Authors:** Jawad Ahmad, Mohamad Ghaffarian Niasar

**Affiliations:** High Voltage Technologies, Department of Electrical Sustainable Energy, Delft University of Technology, 2628 CD Delft, The Netherlands

**Keywords:** perfluoroalkoxy alkane (PFA), aerospace cable insulation, thermal aging, hybrid-electric aircraft, crystallinity, thermomechanical properties, cable bending behavior

## Abstract

The thermal reliability of high-voltage aerospace cables is increasingly important for hybrid-electric and all-electric aircraft; however, most thermal aging studies of perfluoroalkoxy alkane (PFA) have focused on isolated specimens and therefore do not establish how material-scale aging translates into the response of a complete cable structure. In this work, a PFA-insulated cable comprising the conductor, primary insulation, and outer jacket was subjected to cyclic thermal aging with a maximum temperature of 280 °C for nominal elapsed durations of up to 1000 h. Each 24 h cycle comprised approximately 1 h of heat-up and 7 h of nominal dwell at 280 °C, followed by natural cooling and recovery. Macroscopic, FTIR, DSC, XRD, TGA, TG–MS, SEM/EDS, contact angle, DMA, tensile, and three-point bending analyses were used to correlate aging of the individual PFA layers with cable-level behavior. The results indicate two dominant aging regimes over the investigated intervals. During the earlier 0–500 h interval, physical annealing and structural ordering predominated, producing increased crystalline organization, morphological densification, restricted molecular mobility, and initial mechanical stiffening. At the later 720–1000 h conditions, degradation-associated structural rearrangement or possible chemi-crystallization became increasingly evident, accompanied by localized morphological defects, partial recovery of molecular mobility, and progressive loss of ductility. The outer jacket exhibited more pronounced aging-related changes in several thermal, morphological, and mechanical responses than the primary insulation. At the cable level, aging was further manifested by axial shrink-back and changes in bending force, secant stiffness, bending work, and apparent structural bending rigidity. The mechanical end-of-life criterion was not reached under the investigated conditions. By linking aging of the individual PFA layers to the dimensional and mechanical response of the assembled cable, this work extends previous material-level PFA aging studies toward cable-scale durability assessment for future electrified aircraft wiring systems.

## 1. Introduction

The increasing electrification of modern aircraft is driven by the need to reduce fuel consumption, emissions, and operating costs while improving overall energy efficiency. In the more-electric aircraft concept, conventional mechanical, hydraulic, and pneumatic systems are progressively replaced by electrical systems, whereas hybrid-electric and all-electric propulsion architectures introduce substantially greater onboard power demands. Future aircraft electrical systems are expected to operate in the megawatt range, with power requirements potentially reaching approximately 40 MW for large electrified aircraft [1]. To transmit such power without excessive conductor mass and Joule losses, electrical architectures are moving toward higher distribution voltages, with propulsion systems potentially reaching approximately 3 kV DC or higher [1,2,3]. These developments impose increasingly severe electrical and thermal stresses on aircraft cabling. Aircraft electrical wiring must additionally operate under severe and highly variable environmental conditions. Depending on installation location and flight condition, aerospace electrical systems may experience temperatures exceeding 200 °C, pressure variations from about 1 to 0.2 atm, changes in humidity, and continuous mechanical vibration [4,5]. Aviation cable specifications commonly require wiring systems to withstand temperatures from approximately −65 °C to at least 150 °C under normal operating conditions [6]. In hybrid-electric propulsion architectures, however, high-power phases such as takeoff and initial climb can impose additional short-duration thermal loads, with cable temperatures potentially approaching approximately 240 °C for 10–15 min depending on the electrical architecture and loading condition [5,7]. Consequently, the long-term reliability of high-temperature cable insulation is becoming a critical consideration in the design of future electrified aircraft power systems.

High-performance polymers used for aerospace electrical insulation include the fluoropolymers polytetrafluoroethylene (PTFE), fluorinated ethylene propylene (FEP), ethylene tetrafluoroethylene (ETFE), and perfluoroalkoxy alkane (PFA), together with other high-temperature engineering polymers such as polyether ether ketone (PEEK) and polyimide (PI) [2,8]. Among these materials, PTFE provides excellent dielectric properties, chemical inertness, and thermal stability but possesses an extremely high melt viscosity that restricts conventional melt extrusion and commonly necessitates tape-wrapped cable constructions [2]. Such architectures may contain small interfacial gas cavities that become particularly undesirable at the elevated electric fields and reduced pressures encountered in high-voltage aerospace systems. PFA retains the highly fluorinated molecular structure and many of the desirable dielectric, chemical, and thermal characteristics of PTFE while offering considerably improved melt processability, enabling the insulation to be directly extruded around the conductor [1,9]. Its high thermal endurance, low dielectric loss, chemical resistance, and continuous service capability at elevated temperature therefore make PFA particularly attractive for emerging high-voltage aerospace cable systems.

Despite its high thermal stability, prolonged exposure of PFA to temperatures approaching its upper operating range can progressively modify its structure and functional properties. More broadly, thermo-oxidative aging studies of aerospace insulation materials such as PEEK, PTFE, and PI have demonstrated that elevated-temperature exposure can produce substantial changes in morphology, thermal behavior, molecular mobility, mechanical performance, and dielectric properties [8]. In our previous material-level investigation, free PFA specimens (PFA films) were thermally aged at 280 °C for up to 1000 h, revealing a progressive evolution involving structural rearrangement and crystallinity changes followed by degradation-related phenomena, together with corresponding changes in thermal, dynamic mechanical, tensile, and dielectric properties [5]. A recent long-term investigation of PFA films has similarly demonstrated that accelerated thermal exposure causes measurable evolution of the physicochemical structure and thermomechanical and electrical characteristics of PFA [4]. These studies provide an important understanding of the intrinsic thermal aging mechanisms of PFA; however, they predominantly characterize the polymer as an isolated material rather than as part of the complete cable structure.

This distinction is important because an operational cable is not simply a free polymer specimen but a geometrically constrained, multilayer thermomechanical system comprising the metallic conductor, primary insulation, and outer protective jacket. During prolonged thermal exposure, relaxation of processing-induced orientation, crystalline rearrangement, dimensional shrinkage, and degradation-related changes occur while the polymer layers remain constrained by the conductor and adjacent interfaces. Consequently, isolated-film aging cannot fully reproduce cable-specific phenomena such as axial shrink-back, interlayer constraint, differential responses of the primary insulation and outer jacket, or changes in whole-cable flexibility and bending resistance. Although previous work has investigated thermally aged PFA cable specimens and reported selected thermal and electrical characteristics [10], a comprehensive relationship between aging of the individual PFA layers and the dimensional, structural, thermomechanical, and mechanical behavior of the complete conductor–insulation–jacket assembly remains insufficiently established. In particular, how material-scale aging processes translate into the bending response and structural integrity of the complete cable requires further investigation.

Building on the material-level understanding obtained in our previous PFA study [5], the present work shifts the focus from intrinsic polymer aging to its manifestation within a complete, mechanically constrained cable structure. A full-scale PFA-insulated aerospace cable comprising the conductor, primary PFA insulation, and outer PFA jacket was subjected to cyclic thermal aging with a maximum temperature of 280 °C for nominal elapsed durations of up to 1000 h. Aging-induced changes were examined through macroscopic and dimensional inspection, FTIR, DSC, XRD, TGA, TG–MS, SEM/EDS, contact angle measurements, DMA, tensile testing, and three-point bending. The addition of XRD and TG–MS provides complementary information on crystalline evolution and degradation-related species, thereby strengthening the structural and mechanistic interpretation. Particular attention was given to differences between the primary insulation and outer jacket, axial shrink-back relative to the conductor, and the relationship between material-scale structural evolution and whole-cable bending behavior. Thus, the principal novelty of the present study lies not in proposing an entirely independent aging mechanism for PFA, but in translating material-scale aging into cable-level dimensional, structural, and mechanical response, supported by a broader characterization framework. This material-to-cable correlation provides aging indicators that are more directly relevant to the durability assessment and design of high-voltage wiring systems for future hybrid-electric and all-electric aircraft.

## 2. Materials and Methods

### 2.1. Cable Material and Construction

The high-voltage PFA-insulated aerospace cable investigated in this study was supplied by GKN Fokker, Papendrecht, The Netherlands. The cable consisted of a nickel-plated copper conductor surrounded by two extruded PFA layers: a primary insulation layer with a thickness of approximately 0.6 mm and an outer jacket with a thickness of approximately 0.7 mm. Both the complete cable assembly and individual PFA layers extracted from the cable were investigated to correlate material-level aging with the response of the complete cable structure.

### 2.2. Accelerated Cyclic Thermal Aging

**Figure 1:**
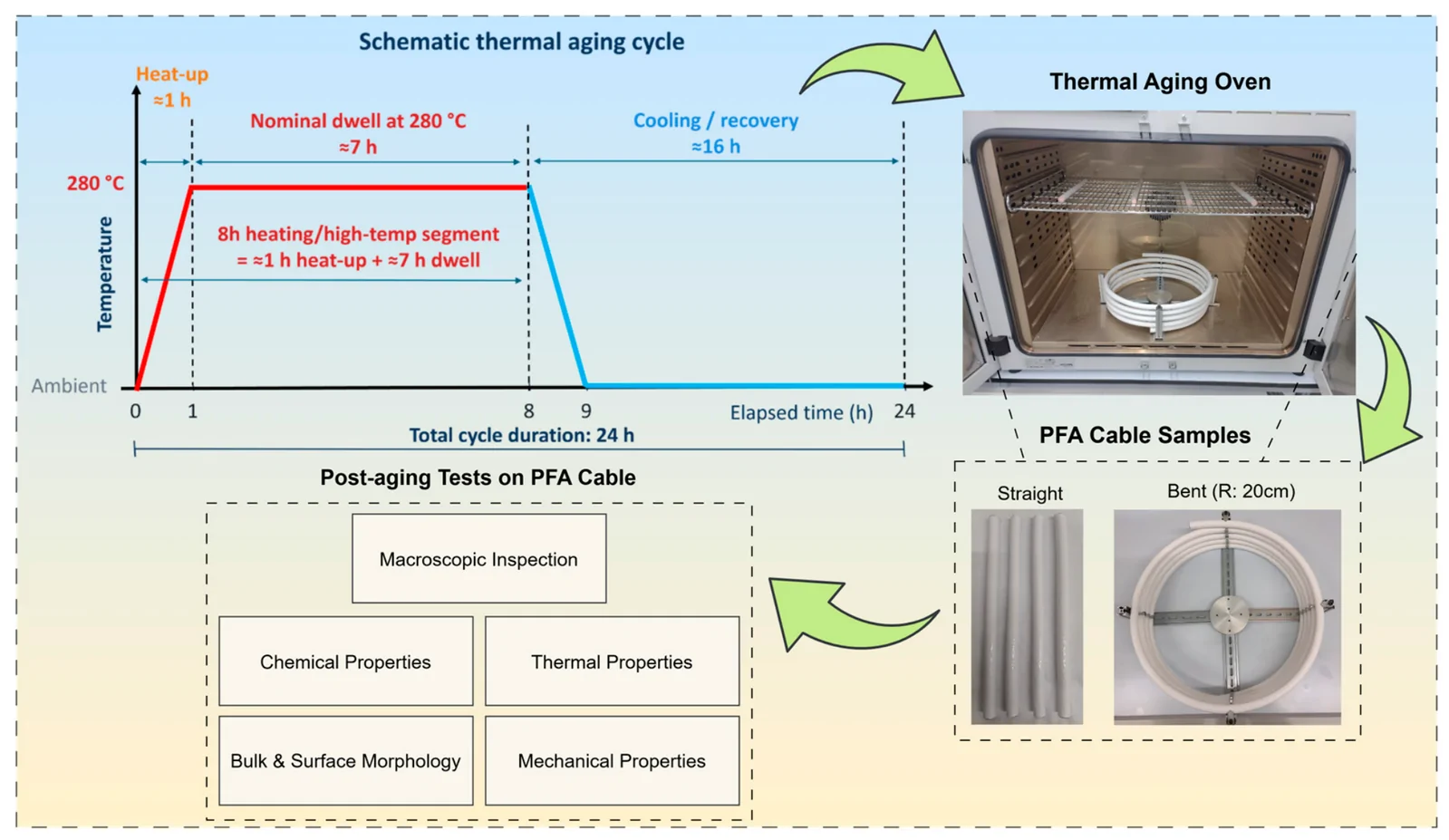
Flowchart for cyclic thermal aging of PFA cable samples.

Prior to thermal exposure, the cable specimens were cleaned with isopropyl alcohol and dried using a lint-free cloth. Straight specimens were cut to a length of 300 mm and positioned on a mesh tray while maintaining a minimum clearance of approximately 100 mm from the oven walls to promote uniform air circulation and avoid direct contact with the heating surfaces. For the wound configuration, cable specimens were wrapped uniformly around a metallic fixture corresponding to the specified minimum bending radius, as illustrated in Figure 1. The specimens were subsequently subjected to cyclic thermal aging with a maximum temperature of 280 °C, which exceeds the nominal continuous service temperature of PFA and was selected to accelerate thermally induced structural evolution.

Each thermal aging cycle had a total elapsed duration of 24 h. The heating/high-temperature segment lasted approximately 8 h and consisted of approximately 1 h of heat-up from ambient temperature to 280 °C followed by approximately 7 h of nominal dwell at 280 °C. The heating system was subsequently switched off, and the specimens underwent natural cooling and recovery during the remaining approximately 16 h of the cycle. Because continuous temperature logging was not performed during the aging campaign, the profile presented in Figure 1 represents the oven operating sequence schematically rather than a directly recorded specimen-temperature history. Specimens were characterized at nominal elapsed cyclic aging times of 240, 500, 720, and 1000 h. These conditions corresponded to 10, 21, 30, and 42 complete thermal cycles, respectively, giving actual elapsed durations of 240, 504, 720, and 1008 h, as shown in Table 1. The corresponding cumulative isothermal dwell times at 280 °C were approximately 70, 147, 210, and 294 h, respectively. For consistency with the experimental sampling schedule and figure labels, the conditions are hereafter referred to as 240, 500, 720, and 1000 h. These labels therefore represent nominal elapsed cyclic aging time rather than continuous exposure at 280 °C. At each designated interval, specimens were removed from the aging program, allowed to equilibrate under ambient conditions, and subsequently subjected to macroscopic, spectroscopic, morphological, thermal, thermomechanical, and mechanical characterization.

### 2.3. Macroscopic Inspection and Axial Shrink-Back Measurement

Macroscopic changes in the cable were documented at each aging condition by comparing the appearance of the straight and wound specimens with that of the unaged cable. Particular attention was given to dimensional changes in the PFA layers relative to the metallic conductor, including axial retraction and conductor exposure at both cable ends.

### 2.4. Fourier Transform Infrared Spectroscopy and 2D Correlation Analysis

Changes in the molecular structure of PFA during aging were investigated using Fourier transform infrared (FTIR) spectroscopy. Spectra were acquired using a PerkinElmer Spectrum 100 spectrometer (PerkinElmer Ltd., Beaconsfield, United Kingdom) in transmission mode over the wavenumber range of 4000–400 cm^−1^. Each spectrum was obtained from 20 accumulated scans at a spectral resolution of 4 cm^−1^. A background spectrum was recorded before each measurement and automatically subtracted to minimize contributions from the surrounding atmosphere. FTIR measurements were performed on two independent specimens for each aging condition to verify the reproducibility of the spectral changes, and one representative spectrum from each condition is presented. To resolve subtle aging-dependent changes in overlapping FTIR bands, synchronous two-dimensional correlation spectroscopy (2D-COS) was performed using aging time as the external perturbation variable. Prior to analysis, the spectra were restricted to the selected regions of interest where the most pronounced aging-related changes were observed. For each aging stage, the corresponding spectra were mean-centered and dynamic spectra were generated by subtracting the stage-averaged spectrum from each individual spectrum as:

(1) $$\tilde{A}\left(\nu ,t\right)=A\left(\nu ,t\right)-\bar{A}\left(\nu \right)$$

where $\tilde{A}\left(\nu ,t\right)$ is the absorbance at wavenumber ν and aging time t, and $\bar{A}\left(\nu \right)$ is the mean spectrum of the corresponding aging stage. The synchronous correlation map was then obtained from the covariance of the dynamic spectra to identify bands whose intensities changed concurrently with aging. The analysis was performed separately for the early and prolonged aging stages to distinguish the dominant spectral evolution in each regime.

### 2.5. Thermal Characterization

#### 2.5.1. Differential Scanning Calorimetry and Thermogravimetric Analysis

Differential scanning calorimetry (DSC) and thermogravimetric analysis (TGA) were employed to characterize aging-induced changes in the crystalline structure and thermal stability of the PFA layers. DSC measurements were performed using a TA Instruments Discovery DSC 250 system (TA Instruments, New Castle, DE, USA), whereas thermogravimetric measurements were conducted using a PerkinElmer TGA 4000 (PerkinElmer, Shelton, CT, USA). For both analyses, specimens with a mass of approximately 8–10 mg were tested under a nitrogen atmosphere at a heating rate of 10 °C min. For both DSC and TGA, measurements were performed on two independent specimens for each aging condition; the replicate measurements showed consistent thermal behavior, and one representative trace is presented for clarity. During DSC measurements, the specimens were heated from room temperature to 350 °C and subsequently cooled to ambient temperature at 10 °C min. Melting and crystallization characteristics were determined from the corresponding heating and cooling thermograms. For TGA, specimens were heated from room temperature to 700 °C at 10 °C min, and the resulting mass loss curves were used to evaluate the thermal decomposition behavior of PFA. For PFA DSC, the percent crystallinity $X_{c}\left(\%\right)$ from DSC melting enthalpy is typically calculated as:

(2) $$X_{c}\left(\%\right)=\frac{{\Delta H}_{m}}{{\Delta H}_{m}^{0}}\times 100$$

where ${\Delta H}_{m}$ is the measured melting enthalpy of the PFA sample, and ${\Delta H}_{m}^{0}$ is the melting enthalpy of 100% crystalline PFA (67 J/g) [5]. The lamellar thickness ($l_{c}$) was also estimated using the modified Gibbs–Thomson relationship [11]. Because PFA is a copolymer of tetrafluoroethylene and perfluoroalkyl vinyl ether, its equilibrium melting temperature differs from that of PTFE and depends on the comonomer concentration. The equilibrium melting temperature of PFA, $T_{m}^{0\prime }$, was therefore estimated according to:

(3) $$T_{m}^{0\prime }={\left[\frac{1}{T_{m}^{0}}+\frac{R}{{\Delta H}_{f}^{0}}X_{P}\right]}^{-1}$$

where $T_{m}^{0}$ is the equilibrium melting temperature of PTFE (616.5 K), R is the universal gas constant (8.314 J mol^−1^ K^−1^), ${\Delta H}_{f}^{0}$ is the molar melting enthalpy of the C_2_F_4_ unit (5.73 kJ mol^−1^), and $X_{P}$ is the molar fraction of PPVE. Since the exact comonomer concentration of the proprietary modified PFA used in the cable was not disclosed by the manufacturer, a PPVE concentration of 1.2 mol% was adopted based on the PFA composition reported in the reference study [11]. The apparent lamellar thickness $I_{c}$ was subsequently determined from:

(4) $$l_{c}=\frac{2\sigma_{e}T_{m}^{0\prime }\Delta z}{{\Delta H}_{f}^{0}\left(T_{m}^{0\prime }-T_{m}\right)}$$

where $\sigma_{e}$ is the folding-surface free energy (2.91 kJ mol^−1^), Δz is the length of the C_2_F_4_ unit (0.254 nm), and $T_{m}$ is the experimentally measured melting peak temperature. The calculated values should therefore be regarded as apparent lamellar thicknesses based on the assumed PPVE concentration rather than direct measurements of lamellar dimensions.

#### 2.5.2. X-Ray Diffraction Analysis

X-ray diffraction (XRD) analysis was performed on PFA samples using a Rigaku MiniFlex 600 X-ray diffractometer (Rigaku Corporation, Akishima, Tokyo, Japan) with Cu Kα radiation (λ=1.5406 Å). Diffraction patterns were collected over a 2θ range of approximately 5–60°. The diffraction profiles were background-corrected, and the characteristic crystalline reflections were analyzed using a consistent fitting procedure to determine their peak positions and full width at half maximum (FWHM). The interplanar spacing, d, was calculated from the corresponding diffraction angle using Bragg’s law:

(5) $$d=\frac{\lambda }{2\sin\theta }$$

Assuming first-order diffraction, the apparent crystallite size along the direction normal to the $\left(100\right)$ planes was estimated from the FWHM of the principal $\left(100\right)$ reflection using the Scherrer equation [12,13]:

(6) $$D_{100}=\frac{K\lambda }{\beta \cos\theta }$$

where K=0.9 is the shape factor, β is the FWHM expressed in radians, λ is the X-ray wavelength and θ is the Bragg angle.

#### 2.5.3. Thermogravimetric–Mass Spectrometric Analysis (TG–MS)

Evolved gases generated during thermal decomposition of the unaged (0 h) and thermally aged (1000 h) PFA insulation were investigated by thermogravimetric analysis coupled to a quadrupole mass spectrometer (QMS 403 C Aëolos, NETZSCH-Gerätebau GmbH, Selb, Germany). Approximately 22 mg of PFA was analyzed under an air atmosphere over the temperature range of 40–1000 °C at a heating rate of 20 °C/min. The evolved gases were transferred continuously from the thermobalance to the mass spectrometer through the heated transfer system. The mass spectrometer was operated in multi-ion detection (MID) mode to selectively monitor characteristic fluorinated and oxygen-containing decomposition products. Ion-current signals were recorded at m/z 20 (HF^+^), 47 (CFO^+^), 50 (CF_2_^+^), 66 (COF_2_^+^), and 69 (CF_3_^+^). The ion-current peak heights were normalized to the initial sample mass (A mg^−1^) to enable direct comparison between the unaged and aged specimens.

### 2.6. Morphological, Elemental, and Surface Wettability Characterization

Aging-induced changes in the surface and bulk morphology of the PFA layers were examined using a JSM-7500F field-emission scanning electron microscope (FE-SEM) (FE-SEM; JEOL Ltd., Akishima, Tokyo, Japan). Prior to imaging, the specimens were sputter-coated with a thin gold layer to minimize charging. SEM observations were performed at an accelerating voltage of 15 kV, and corresponding surface and bulk regions were compared across the different aging conditions. Elemental composition was evaluated using an Oxford Instruments energy-dispersive X-ray spectroscopy (EDS) system (Oxford Instruments NanoAnalysis, High Wycombe, Buckinghamshire, United Kingdom) coupled to the SEM. The relative concentrations of the principal detected elements and the corresponding elemental ratios were compared to evaluate compositional changes associated with prolonged thermal exposure. EDS measurements were performed on two samples for each aging condition to verify the reproducibility of the observed surface-compositional trends. The elemental composition reported corresponds to one representative measurement from each aging condition.

Surface wettability was evaluated through static water contact angle measurements using a KSV CAM 200 optical contact angle system (KSV Instruments Ltd., Helsinki, Finland). A deionized water droplet with a volume of approximately 3 µL was deposited onto the PFA surface, and the equilibrium contact angle was measured using the sessile-drop method. Changes in contact angle with aging were used as an additional indicator of evolution in the surface condition of the PFA layers. Contact angle measurements were performed on two independent specimens for each aging condition, with measurements taken at multiple surface locations, and the reported values represent the average of the replicate measurements.

### 2.7. Thermomechanical and Mechanical Characterization

#### 2.7.1. Dynamic Mechanical Analysis

Dynamic mechanical analysis (DMA) was conducted using a TA Instruments RSA-G2 analyzer (TA Instruments, New Castle, DE, USA) in tension mode. Measurements were performed at a frequency of 1 Hz while the specimens were heated from room temperature to 250 °C under a controlled temperature ramp. The storage modulus and loss factor were evaluated as functions of temperature to determine changes in stiffness, relaxation behavior, and molecular mobility induced by thermal aging. Both the primary insulation and outer PFA jacket were examined to identify differences in their thermomechanical evolution. DMA measurements were performed on two independent specimens for each aging condition, and the replicate measurements exhibited consistent viscoelastic behavior; therefore, one representative curve is presented for each condition.

#### 2.7.2. Tensile Testing

**Figure 2:**
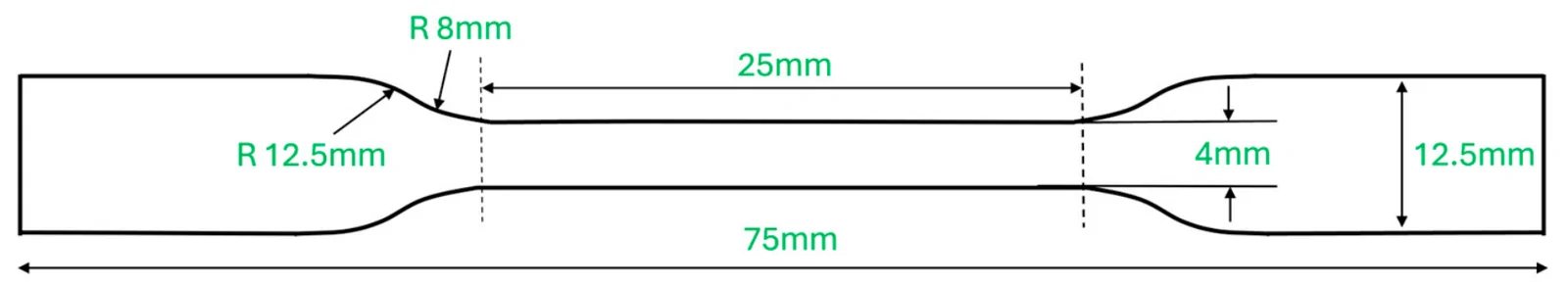
Dumbbell-shaped PFA specimen for tensile testing.

The tensile response of the PFA insulation was evaluated using specimens prepared in a dumbbell geometry by laser cutting, as illustrated in Figure 2. Mechanical testing was carried out at room temperature using a Zwick universal testing machine (ZwickRoell GmbH & Co. KG, Ulm, Germany) equipped with a 1 kN load cell. A constant crosshead speed of 10 mm/min was applied. An extensometer attached directly to the gauge region was used to measure specimen strain during loading. Stress–strain curves were obtained for each aging condition and used to determine tensile strength and elongation at break. Five replicate specimens were tested for each condition, and the resulting mean values and associated experimental errors were determined.

#### 2.7.3. Three-Point Bending of the Complete Cable

**Figure 3:**
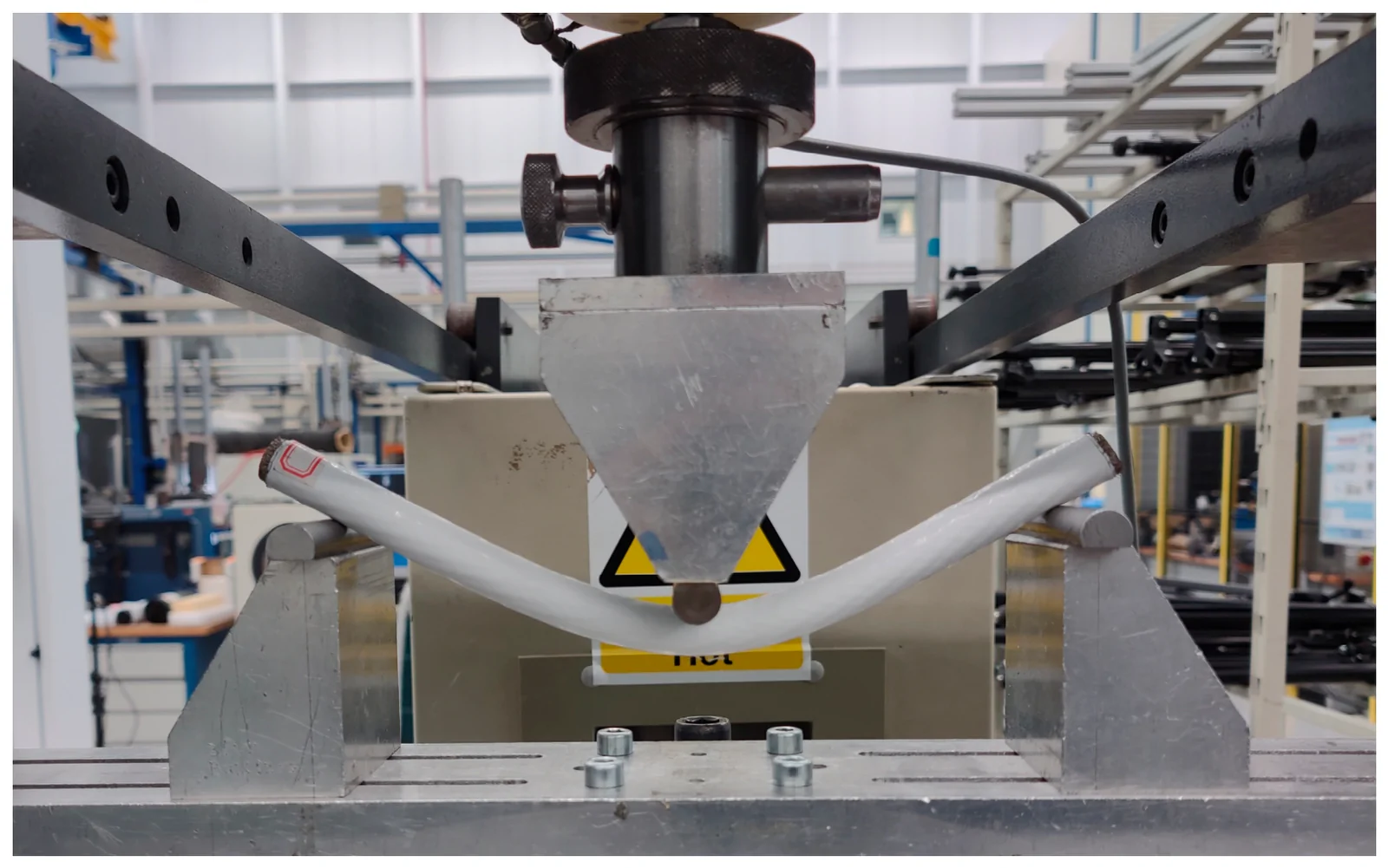
Illustration of PFA cable under the 3PB test.

The cable-scale mechanical response was evaluated using a modified three-point bending (3PB) configuration. Measurements were performed using a Zwick universal testing machine (ZwickRoell GmbH & Co. KG, Ulm, Germany) equipped with a 20 kN load cell under displacement control at a crosshead speed of 5 mm/min. Both the complete PFA cable and the corresponding jacket-removed configuration were tested to distinguish the contribution of the outer PFA jacket from that of the conductor–primary-insulation assembly. The complete cable had an outside diameter of 17.4 mm. For each aging condition, two independent specimens were tested. Cable specimens with a total length of 30 cm were placed on two cylindrical supports with a support span, L, of 225 mm and loaded centrally under displacement control, as shown in Figure 3. Both the lower supports and the central loading nose had a radius of 7.5 mm. Force and crosshead displacement were continuously recorded up to the prescribed maximum displacement, δ of 45 mm. Because the cable is a heterogeneous multilayer structure and undergoes relatively large deformation during bending, the assumptions of the homogeneous small-deflection beam theory are not strictly applicable. However, the initial portion of the force–displacement response was approximately linear and was used to obtain an apparent structural bending rigidity for comparative purposes. The slope, m, of the selected initial linear region was determined from the force–displacement curve, and the corresponding apparent bending rigidity was calculated as:

(7) $$EI_{app}=\frac{mL^{3}}{48}$$

where L is the support span. The resulting $EI_{app}$ is reported only as a comparative structural parameter describing the initial bending resistance of the complete cable assembly and should not be interpreted as an intrinsic flexural property of the constituent PFA material. To provide a common deformation level relevant to the cable installation constraint, the force response was additionally evaluated at a crosshead displacement corresponding geometrically to the recommended minimum bending radius of 20 cm. Assuming an ideal circular arc with radius R = 200 mm spanning a chord equal to the support distance L=225 mm, the corresponding midspan sagitta, $\delta_{R20}$, was estimated as

(8) $$\delta_{R20}=R-\sqrt{R^{2}-{\left(\frac{L}{2}\right)}^{2}}$$

Using a circular arc geometrical approximation gave an equivalent midspan displacement, $\delta_{R20}$ of 34.6 mm. The value was used only as a geometrical reference displacement and does not imply that the cable attained a uniform or experimentally verified local bending radius of exactly 200 mm. The force at this displacement, $F_{R20}$, was obtained directly from the force–displacement curve. A corresponding secant stiffness was calculated as:

(9) $$K_{sec,R20}=\frac{F_{R20}}{\delta_{R20}}$$

The mechanical work required to bend the cable from the undeformed condition to the equivalent 20 cm bending radius condition, $U_{R20}$, was determined from the area under the force–displacement curve:

(10) $$U_{R20}=\int_{0}^{\delta_{R20}}F\left(\delta \right)\;d\delta$$

The differences between the full-cable and jacket-removed configurations were used to quantify the incremental contribution of the outer PFA jacket. These parameters were used to relate aging-induced changes in the individual PFA layers to the mechanical response and flexibility of the complete cable assembly.

## 3. Results and Discussion

### 3.1. Macroscopic Evolution and Axial Shrink-Back Analysis

**Figure 4:**
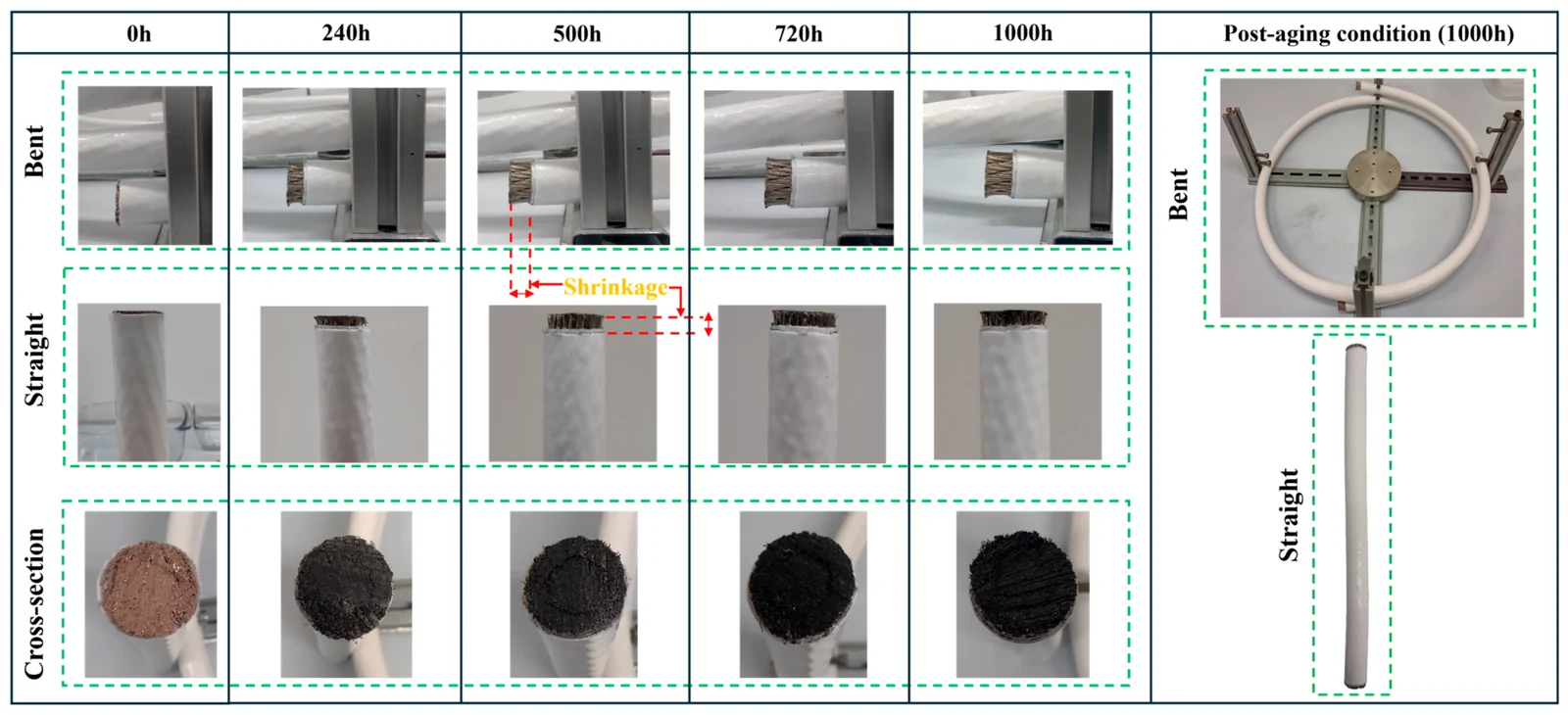
Macroscopic evolution of straight and bent PFA cables before and after thermal aging.

Figure 4 presents the macroscopic appearance of the straight and wound PFA-insulated cables before and after thermal aging. In the unaged cable, the PFA layers were approximately aligned with the conductor ends. Thermal aging produced progressive exposure of the conductor at both ends, indicating axial shrink-back (also termed as insulation retraction) of the polymer layers. For the straight specimens, the total axial shrink-back, $S_{a}\left(\%\right)$ was calculated from the retraction measured at both ends:

(11) $$S_{a}\left(\%\right)=\frac{{\Delta L}_{left}+{\Delta L}_{right}}{L_{0}}\times 100$$

where ($L_{0}$) is the initial specimen length ($L_{0}$ = 30 cm), while ${\Delta L}_{left}$ and ${\Delta L}_{right}$ are the retractions measured at the two ends. For the 300 mm straight specimens, the end retraction increased from approximately 3 mm/end at 240 h to 4 mm at 500 h, 4–5 mm at 720 h, and approximately 5 mm at 1000 h, as described in Table 2. Assuming approximately equal retraction at both ends, these values correspond to total axial shrink-back of 2.00%, 2.67%, 2.67–3.33%, and approximately 3.33%, respectively. The largest dimensional change occurred during the early aging period, followed by a progressively smaller increase, indicating an approach toward dimensional stabilization. The bent cable also exhibited visible terminal retraction, with measured values of approximately 5–6 mm at 240 h, 7–8 mm at 500 h, 9–10 mm at 720 h, and approximately 10 mm at 1000 h. The substantially greater length of the bent specimen may have contributed to the larger absolute end retraction by allowing dimensional recovery to accumulate over a longer polymer length. However, these values were not directly compared with those of the straight specimens because the experimental configurations differed substantially. The bent specimen was initially a 5 m wound cable, from which a 1.25 m section was removed after each inspection. Consequently, the remaining cable length decreased progressively, and each sectioning step created a new free-end boundary condition. The measured values for the bent configuration therefore represent checkpoint-specific local end retraction rather than cumulative axial shrinkage of a fixed-length specimen. Accordingly, no bent-to-straight shrinkage ratio was calculated, and the independent effect of bending on axial shrink-back cannot be quantitatively isolated from the present experimental configuration. Nevertheless, the wound geometry imposed a mechanically strained condition on the cable. For a cable diameter of D = 17.4 mm and a bending radius of $R_{b}$ = 200 mm, the nominal outer-fiber bending strain can be estimated as:

(12) $$\varepsilon_{b}\approx \frac{D}{2R_{b}}\approx \frac{17.4}{2\left(200\right)}\times 100\%\approx 4.35\%$$

This value indicates the presence of additional tensile and compressive strain on the outer and inner sides of the wound cable, respectively. However, because specimen length, free-end conditions, and sectioning history differed from those of the straight specimens, Equation 12 is used only to characterize the imposed bending geometry and not to quantify an enhancement of shrinkage due to bending. The outer jacket retracted approximately 0.5–0.8 mm farther than the primary insulation, as evident from Figure 4, which may be associated with its lower mechanical constraint, greater distance from the cable neutral axis and different extrusion draw-down history. The observed shrink-back is primarily attributed to relaxation of the molecular orientation and residual stresses introduced during extrusion and draw-down. During extrusion, PFA chains become preferentially oriented along the cable axis, and part of this orientation is retained when the polymer solidifies. The aging temperature of 280 °C lies within the reported sub-melting annealing region of PFA and remains below its typical melting range of approximately 300–310 °C [11,14]. Under these conditions, PFA polymer possesses sufficient molecular mobility within the amorphous regions that can facilitate relaxation of the oriented chains, crystalline rearrangement, lamellar thickening and irreversible axial recovery [11]. Because the copper conductor and interlayer contact restrict dimensional recovery along the cable length, the contraction becomes most apparent at the unconstrained ends. The difference in thermal expansion between the PFA layers and the copper conductor may additionally generate interfacial stresses and promote limited relative displacement during thermal cycling.

Despite the axial shrink-back, no changes in cable outer diameter or total mass were detectable within the measurement resolution, as shown in Table 2. The PFA retained its original color, although slight surface dulling was observed after 720 and 1000 h. No macroscopic cracking or radial delamination was visible. Darkening of the exposed conductor strands and cross-sectional surface was observed after aging; this appearance is suggestive of surface oxidation but cannot establish the oxide composition from visual inspection alone. Overall, the visual and dimensional observations indicate two overlapping stages. The early stage, extending approximately to nominal elapsed duration of 500 h, was dominated by sub-melting annealing, release of extrusion-induced residual stress, rapid axial shrink-back and an approach toward dimensional stabilization. During the later stage, the retraction rate decreased as the available residual stress and molecular orientation were progressively exhausted. The limited additional retraction and slight surface dulling observed at longer aging durations may reflect slower morphological evolution and/or incipient surface degradation; these possibilities are further examined using SEM/EDS, FTIR, and thermal analyses in subsequent sections.

### 3.2. Chemical Structure Evolution

#### 3.2.1. FTIR Analysis

**Figure 5:**
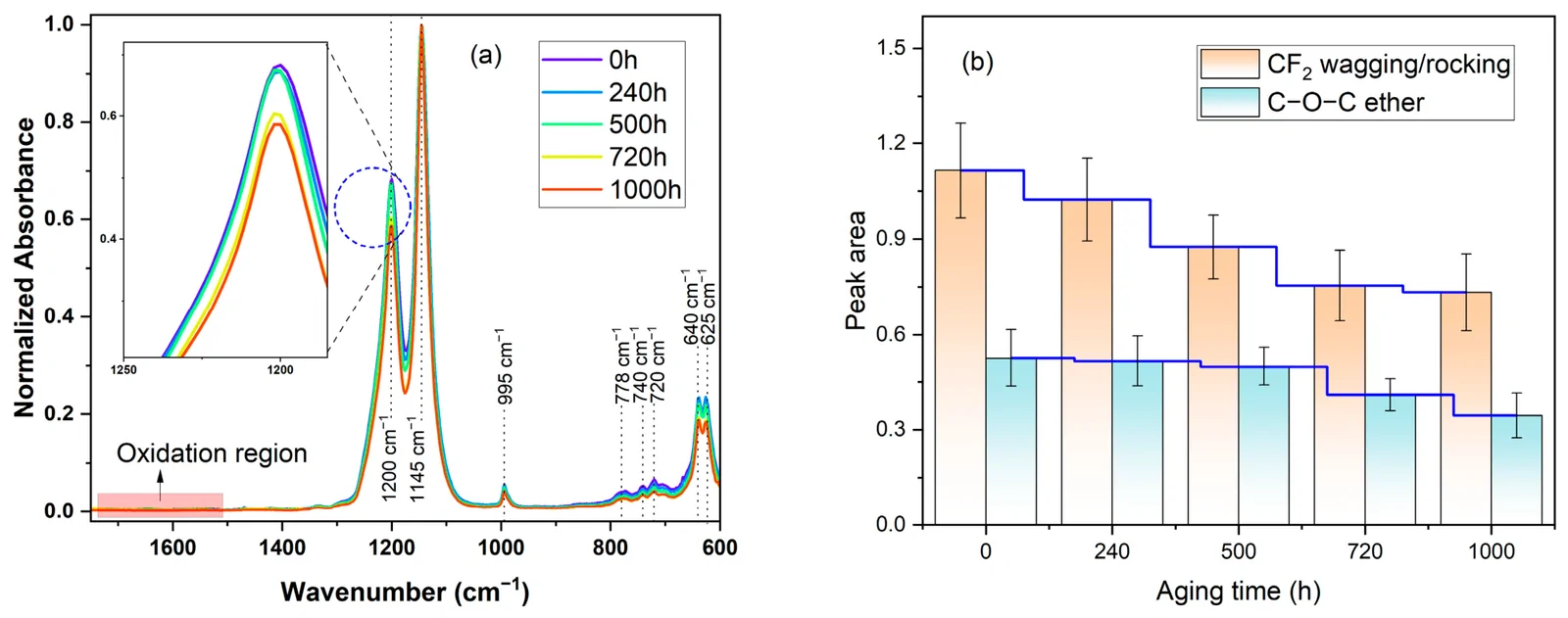
(**a**) Normalized FTIR spectra of PFA insulation, (**b**) peak area variation in CF_2_ wagging/rocking and C–O–C ether vibration bands during thermal aging.

To facilitate comparison of aging-induced spectral changes, the FTIR spectra were normalized to the maximum absorbance of each spectrum, which corresponded to the dominant CF_2_ backbone band and served as a common reference for comparing weaker aging-sensitive bands (Figure 5a) [15]. The unaged PFA exhibits strong absorption bands in the range of 1145–1200 cm^−1^, which are mainly attributed to C–F stretching vibrations of the CF_2_ backbone and CF_3_-containing side-chain groups [5,16]. A distinct absorption band around 995 cm^−1^ is assigned to the C–O–C stretching vibration associated with ether-containing perfluoroalkoxy side groups [17]. In addition, the characteristic peaks observed at approximately 720, 740, and 778 cm^−1^ are mainly related to CF_2_ wagging and rocking vibrations, while contributions from CF_3_ bending vibrations may also appear in this region [5,18,19]. These bands are sensitive to chain packing and the amorphous/crystalline phase structure of PFA [5]. The absorption bands in the 600–650 cm^−1^ region are assigned to CF_2_ bending vibrations.

The spectra show that the overall FTIR features remain largely unchanged during the early aging stages from 0 to 500 h, with the characteristic fluorocarbon absorption bands of PFA remaining clearly preserved and no major new absorption features appearing. In particular, the relative intensity of the band near 1200 cm^−1^ remains almost unchanged at the early aging stages, suggesting that obvious chemical degradation does not occur. This indicates that the main fluorocarbon backbone remains largely intact during the early aging period; however, more noticeable aging-dependent changes were observed in the weaker characteristic bands. The bands at 720, 740, and 778 cm^−1^ show a gradual decrease in relative intensity with increasing aging time, as shown in Figure 5a. Since these bands are associated with CF_2_ wagging/rocking modes and are sensitive to phase organization, their variation suggests changes in local molecular conformation and chain packing. The reduction in these amorphous-phase-sensitive bands may indicate a decrease in the amorphous contribution, which can be related to increased chain ordering and crystallinity during the thermal aging process [4,5,8]. This supports the interpretation that physical annealing and structural rearrangement are dominant during the early stage of aging. The integrated peak area analysis presented in Figure 5b provides additional evidence for this stage-dependent evolution. The integrated area of the CF_2_ wagging/rocking region decreases progressively from the unaged condition through 500–720 h, whereas only a comparatively small additional decrease is observed between 720 and 1000 h. The reduced rate of change at prolonged exposure suggests that the conformational rearrangement of the CF_2_ segments is approaching stabilization, indicating that a substantial fraction of the annealing-driven chain reorganization has already occurred.

More pronounced chemical changes become evident after prolonged aging, particularly at 720 and 1000 h. The relative intensity of the band near 1200 cm^−1^ decreases noticeably, as highlighted in the enlarged inset of Figure 5a, indicating changes in the local CF_2_/CF_3_ environment and suggesting modification or degradation of fluorinated side-chain units. Similar chemical evolution during prolonged thermal aging of PFA has been reported by Henri et al. [4]. Peacock et al. reported that changes in crystallinity can induce frequency shifts in CF_2_ absorption bands, particularly around 1145 cm^−1^; however, no appreciable shift in this band is observed in the present spectra [20]. The evolution of the ether-related C–O–C band near 995 cm^−1^ further distinguishes the prolonged aging stage. As shown by the integrated peak areas in Figure 5b, the C–O–C contribution changes only slightly during the earlier aging period but exhibits a more pronounced reduction at 720–1000 h. Importantly, this decrease continues while the CF_2_ wagging/rocking peak area begins to approach stabilization. The different temporal evolution of these two spectral regions suggests that, once much of the initial conformational rearrangement has occurred, chemical modification of the ether-containing perfluoroalkoxy side-chain structure becomes increasingly important. Moreover, the changes in the CF_3_/side-chain and ether-related bands become more pronounced after approximately 500 h, whereas the CF_2_ bending and wagging/rocking modes undergo comparatively smaller changes at the longest aging times. This suggests that the ether-containing and fluorinated side-chain units are more sensitive to prolonged thermal exposure than the main CF_2_ framework.

No obvious new absorption bands associated with oxidation products, such as carbonyl groups or unsaturated fluorinated degradation species, are observed in the examined oxidation-sensitive region. This indicates that PFA still retains good thermo-oxidative stability after aging at 280 °C for a nominal elapsed duration of 1000 h. However, the gradual weakening of the ether and side-chain-related bands at prolonged aging times suggests the onset of molecular-level degradation. Chain scission of side-chain or ether-containing structures may generate shorter molecular segments, which can subsequently rearrange into more ordered regions [5]. Therefore, the FTIR results suggest that early-stage aging of PFA is mainly governed by physical annealing and structural ordering, whereas long-term aging, particularly at 720–1000 h, involves degradation-assisted rearrangement or possible chemi-crystallization.

#### 3.2.2. Stage-Resolved Synchronous (2D-COS) Analysis

**Figure 6:**
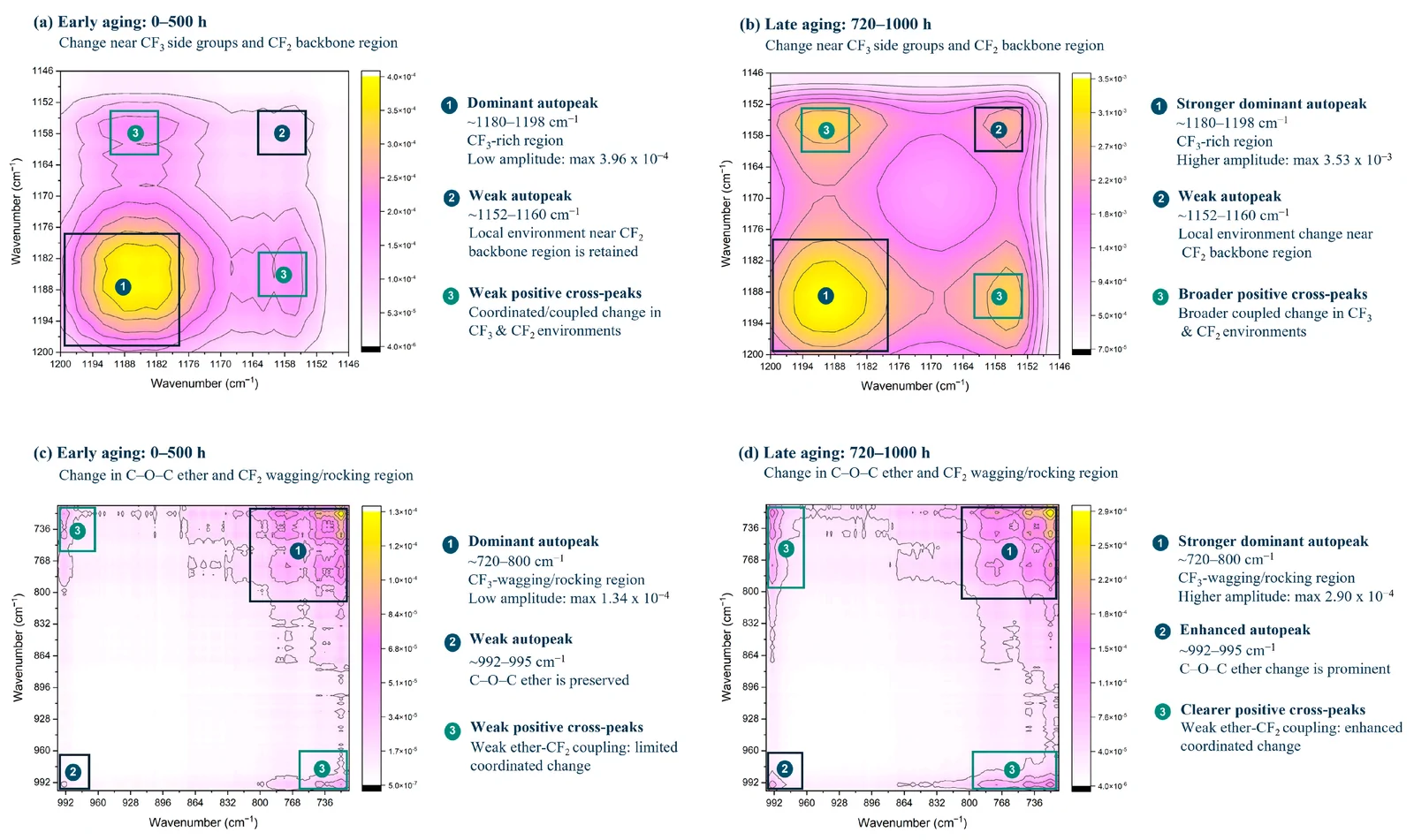
Stage-resolved synchronous two-dimensional correlation maps of PFA during thermal aging: (**a**) CF_3_ side-group and CF_2_ backbone region during early aging from 0 to 500 h; (**b**) CF_3_ side-group and CF_2_ backbone region during prolonged aging from 720 to 1000 h; (**c**) C–O–C ether and CF_2_ wagging/rocking regions during early aging; and (**d**) the corresponding ether and CF_2_ regions during prolonged aging.

Stage-resolved synchronous two-dimensional correlation analysis was conducted on FTIR spectra as a complementary technique to further clarify the relative evolution of the characteristic PFA vibrational bands during the early and prolonged thermal aging stages, as shown in Figure 6. In these maps, diagonal auto-peaks represent the spectral variance of individual bands, whereas symmetric off-diagonal cross-peaks indicate coordinated intensity changes between different vibrational regions.

During the early aging stage from 0 to 500 h, Figure 6a shows relatively low covariance within the 1145–1200 cm^−1^ C–F stretching region. The largest early-stage auto-peak occurs around 1186–1190 cm^−1^, within the higher-wavenumber CF_3_ associated C–F environment. However, the maximum synchronous intensity is only $3.96\times 10^{-4}$, confirming that the corresponding variation remains small. A weaker response occurs around 1155–1165 cm^−1^, while the weak positive cross-peaks indicate limited coordinated spectral redistribution between CF_2_ and CF_3_ associated C–F stretching vibrations. These results agree with the FTIR spectra, which showed that the main fluorocarbon absorption features remain largely preserved during early aging. Consequently, the early-stage covariance should be attributed mainly to small changes in molecular conformation, chain packing, orientation and crystalline/amorphous phase distribution. During prolonged aging from 720 to 1000 h, Figure 6b shows a substantially stronger response in the C–F stretching region, with a maximum synchronous intensity of $3.53\times 10^{-3}$. The dominant variation remains centered near 1188–1192 cm^−1^, while a more noticeable auto-peak appears around 1152–1160 cm^−1^. The stronger positive cross-peaks indicate that changes in the higher-wavenumber CF_3_ environment become increasingly coupled with changes in the local CF_2_ environment. However, the persistence of the characteristic C–F absorption pattern and the absence of new degradation-related bands show that the fluorocarbon backbone remains largely intact. The enhanced covariance therefore indicates increasing perturbation of the local CF_2_/CF_3_ environments.

A clearer distinction between the two aging stages is obtained from the maps covering the approximately 720–995 cm^−1^ region as highlighted in Figure 6c,d. During early aging (Figure 6c), the spectral response is concentrated primarily in the 720–800 cm^−1^ region associated with CF_2_ wagging and rocking vibrations. The approximately 995 cm^−1^ C–O–C ether auto-peak remains weak, and only limited cross-correlation is observed between the ether and CF_2_ conformational modes indicating that the ether-containing side groups remain comparatively unaffected during this stage. Because the 720, 740 and 778 cm^−1^ bands are sensitive to molecular conformation and amorphous/crystalline phase organization, their early variation indicates thermally induced rearrangement of the polymer chains [5]. This supports physical annealing, improved packing and increased structural ordering as the dominant early-stage processes [11]. During prolonged aging, Figure 6d shows that the approximately 995 cm^−1^ ether response becomes more distinct and the symmetric cross-peaks between the ether vibration and the 720–800 cm^−1^ CF_2_ conformational modes become clearer. This indicates increasing involvement of the ether-containing perfluoroalkoxy side groups during long-term thermal exposure. Meanwhile, the continued response of the CF_2_ wagging and rocking modes demonstrates that phase reorganization and chain packing remain active. The combined spectral response is therefore consistent with mild degradation of ether/side-chain structures producing shorter and more mobile molecular segments that subsequently participate in further ordering and crystallization.

Overall, the synchronous correlation maps further support a two-stage aging mechanism observed in FTIR analysis. The early stage is dominated by physical annealing, local conformational rearrangement and increased chain ordering. During prolonged aging, limited degradation of the ether-containing and fluorinated side-chain environments becomes coupled with continuing phase reorganization, resulting in possible degradation-assisted chemi-crystallization. The absence of new oxidation-related absorption bands and preservation of the principal CF_2_ stretching features demonstrate that severe degradation of PFA does not occur after exposing it to thermal aging profile for a nominal elapsed duration of 1000 h at 280 °C.

### 3.3. Crystalline Evolution and Thermal Stability

#### 3.3.1. DSC and Crystallinity Evolution

**Figure 7:**
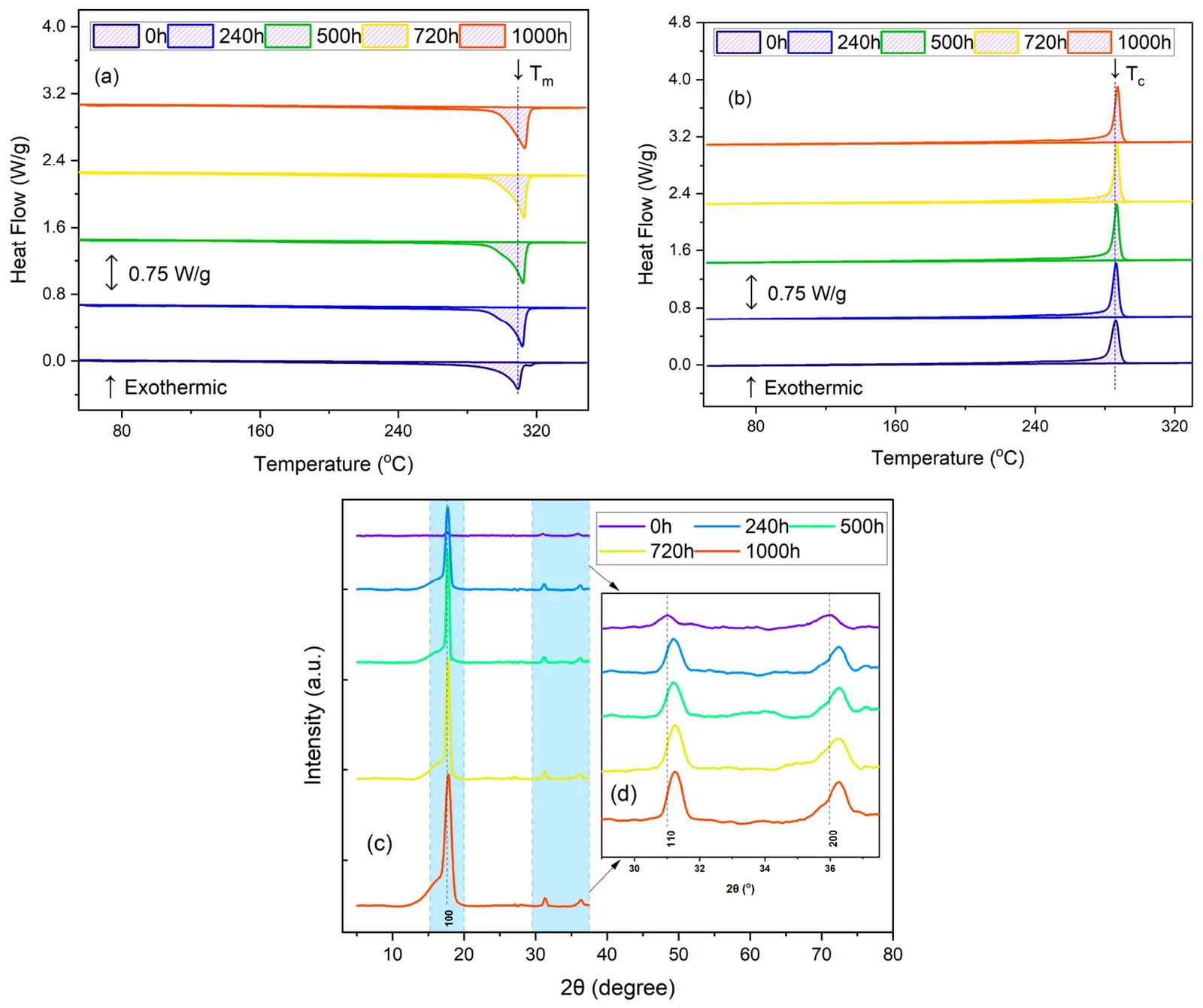
DSC and XRD curves of PFA insulation: (**a**) DSC heating. (**b**) DSC cooling. (**c**) Evolution of the XRD patterns. (**d**) (110) and (200) Crystalline reflections of PFA during thermal aging.

The evolution of the crystalline structure of PFA during thermal aging was further evaluated from the melting behavior, crystallinity, and apparent lamellar thickness. The DSC melting and crystallization curves of PFA during aging evolution are shown in Figure 7. The DSC results in Table 3 indicate distinct stages of structural evolution during thermal aging. During the first stage, from 0 to approximately 500 h, the behavior was predominantly governed by thermal annealing. The melting onset increased from 301.6 to 304.2 °C, while the melting peak temperature increased from 309.2 to 312.1 °C. At the same time, the melting enthalpy increased markedly from 30.4 to 37.8 J/g, corresponding to an increase in crystallinity from 45.3 to 56.4%. The full width at half maximum (FWHM) of the melting peak decreased from 8.39 to 7.89 °C, indicating a relatively narrower distribution of crystal stability as shown in Figure 7a. In parallel, the calculated apparent lamellar thickness increased from 5.70 to 6.37 nm, corresponding to an increase of approximately 11.7%. These simultaneous increases in crystallinity, melting temperature, melting onset, and lamellar thickness, together with narrowing of the melting endotherm, are consistent with an annealing-dominated process involving molecular rearrangement, lamellar thickening, and improvement in crystal ordering [11,21,22,23]. These changes in PFA indicate progressive crystal perfection during the early aging stage (0–500 h), where sub-melting thermal exposure facilitates the rearrangement of crystallizable chain segments within the amorphous regions and their incorporation into existing or newly developed lamellae, driving the structure toward a more thermodynamically stable crystalline state [21,22,23].

Beyond approximately 500 h, the crystalline evolution of PFA changed considerably. The increase in crystallinity became very limited, rising only from 56.4% at 500 h to 57.1% at 1000 h, while the apparent lamellar thickness increased only moderately from 6.37 to 6.61 nm as shown in Table 3. Similarly, the melting peak temperature increased by only 0.9 °C, from 312.1 to 313.0 °C, indicating that further lamellar thickening or crystal perfection became comparatively limited in PFA. In contrast, the melting behavior became increasingly heterogeneous. The FWHM increased from 7.89 °C at 500 h to 9.17 °C at 1000 h, while the melting onset ultimately decreased to 302.0 °C despite the slight continued increase in the principal melting peak temperature. The simultaneous broadening of the melting endotherm, reduction in melting onset, and only minor increase in the principal melting temperature suggest the development of a broader population of crystalline structures in PFA with different thermal stabilities. This behavior is consistent with a transition from predominantly physical annealing to degradation-assisted chemi-crystallization. During prolonged thermal exposure of PFA, limited chain scission may generate shorter and more mobile molecular segments capable of reorganizing into additional crystalline regions [5,24,25]. These newly formed crystals may be thinner and/or less perfect than the pre-existing annealed lamellae, thereby contributing to the lower-temperature portion of the melting endotherm and reducing $T_{onset}$. At the same time, the persistence and slight further thickening of more stable pre-existing lamellae can account for the modest increase in the principal $T_{m}$ and calculated $I_{c}$. Consequently, the broader FWHM observed at prolonged aging reflects an increasingly heterogeneous lamellar population rather than simple continuous crystal perfection.

The crystallization behavior of PFA also evolved systematically with thermal aging as shown in Figure 7b. The crystallization peak temperature $T_{c}$ shifted slightly toward higher temperature, from 286.0 °C for the unaged PFA to 287.2 °C after a nominal elapsed duration of 1000 h, while the crystallization exotherms became progressively sharper and more intense. These changes indicate enhanced crystallization kinetics in the aged PFA, likely associated with increased molecular ordering and, at prolonged aging times, greater mobility of shorter chain segments generated during degradation-assisted chemi-crystallization. Since the total $T_{c}$ shift is small, this behavior is best considered supporting rather than standalone evidence of the aging mechanism.

#### 3.3.2. Comparative Crystallinity Evolution of PFA Primary Insulation and Jacket

A supplementary DSC comparison performed in air was used to evaluate differences in the crystallization response of the PFA primary insulation and outer jacket during thermal aging. As shown in Table 4, the unaged jacket exhibited a slightly lower crystallinity (45.5%) than the primary insulation (46.0%), with corresponding melting enthalpies of 30.5 and 30.8 J/g, respectively. This small difference may arise from differences in the extrusion, drawing/orientation, and cooling histories experienced by the two PFA layers during cable manufacture, which can influence their initial molecular organization and crystalline state. During the first 500 h, crystallinity increased markedly in both components, but the relative increase was greater for the jacket. The crystallinity of the primary insulation increased by approximately 19.1%, from 46.0 to 54.8%, whereas that of the jacket increased by approximately 22.0%, from 45.5 to 55.5%. The corresponding increases in ${\Delta H}_{m}$, from 30.8 to 36.7 J/g for the insulation and from 30.5 to 37.2 J/g for the jacket, further indicate enhanced crystalline ordering. The stronger initial crystallization response of the jacket may partly result from its slightly lower initial crystalline state and differences in processing-induced molecular orientation, providing greater potential for chain rearrangement during the annealing-dominated stage. Beyond 500 h, the increase in crystallinity became much smaller for both PFA layers, indicating that most of the annealing-induced structural reorganization had already occurred. Between 500 and 1000 h, the crystallinity increased by approximately 2.0% for the PFA primary insulation and 2.5% for the jacket. The slightly greater late-stage increase observed for the PFA jacket may indicate a somewhat greater susceptibility to degradation-assisted chemi-crystallization, in which degradation-generated shorter chain segments acquire sufficient mobility to reorganize into additional crystalline regions. However, because the difference is relatively small, this result should be considered supportive rather than conclusive evidence of a more pronounced chemi-crystallization process in the PFA jacket.

Over the complete nominal elapsed duration of 1000 h, crystallinity increased by approximately 21.5% for the PFA primary insulation and 25.1% for the jacket. The greater overall crystallinity development of the PFA jacket therefore suggests a larger reduction in amorphous content and molecular mobility, which may contribute to its greater tendency toward stiffening and hardening during prolonged thermal aging.

#### 3.3.3. X-Ray Diffraction Analysis of Crystalline Structure Evolution

Figure 7c,d present the XRD patterns of PFA as a function of thermal aging time, while the corresponding peak positions, crystallinity, interplanar spacing, and apparent crystallite size are summarized in Table 5. All specimens exhibit a dominant crystalline reflection at approximately 2θ=17.5–17.7°, together with weaker reflections at approximately 30.9–31.2° and 35.9–36.3°, assigned here to the $\left(100\right)$, $\left(110\right)$, and $\left(200\right)$ crystalline planes, respectively [11,26,27,28,29]. The persistence of these reflections throughout aging indicates that thermal exposure does not produce a major transformation of the underlying crystalline structure; instead, aging primarily modifies crystal packing, interplanar spacing, and crystalline-domain organization. A pronounced structural rearrangement occurs during the early stage of aging. The $\left(100\right)$ reflection shifts progressively from 17.5° for the unaged PFA to 17.7° after 500 h. According to Bragg’s law, this shift toward higher diffraction angle corresponds to a decrease in the $\left(100\right)$ interplanar spacing from approximately 5.064 Å to 5.007 Å, as shown in Table 5. Similar changes are observed for the higher-order reflections: the $\left(110\right)$ peak shifts from 30.9° to 31.1°, corresponding to a reduction in $d_{110}$ from approximately 2.892 to 2.873 Å, while the $\left(200\right)$ reflection shifts from $35.9^{{}^{\circ}}$ to 36.3°, corresponding to a reduction in $d_{200}$ from approximately 2.500 to 2.473 Å. These relatively small but systematic shifts suggest a modest contraction or tightening of the crystalline lattice, consistent with improved chain packing and relaxation of less-ordered crystalline regions during thermal annealing [11]. The evolution of the $\left(100\right)$ peak width further supports this interpretation. The principal crystalline reflection becomes more pronounced and sharper during the early aging stage, with its FWHM decreasing from 0.58° to 0.50°. Consistent with this peak narrowing, the apparent Scherrer crystallite size increases from approximately 13.9 nm in the unaged specimen to 16.1 nm after 500 h. The simultaneous reduction in interplanar spacing, narrowing of the principal crystalline reflection, and increase in coherent-domain size indicate improved crystalline order and more extended coherent packing during the early aging stage. These structural changes align well with the increase in crystallinity independently observed by DSC, supporting an annealing-dominated mechanism in which elevated-temperature exposure promotes molecular rearrangement and more efficient incorporation of less-ordered chain segments into existing crystalline regions [11].

A distinctly different evolution is observed during prolonged aging. Beyond approximately 500 h, the positions of the $\left(100\right)$, $\left(110\right)$, and $\left(200\right)$ reflections remain essentially unchanged, indicating that the average lattice spacings have largely stabilized. In contrast, the $\left(100\right)$ reflection progressively broadens: the FWHM increases from 0.50° at 500 h to 0.52° at 720 h and 0.79° at 1000 h. Correspondingly, the apparent coherent-domain size decreases from approximately 16.1 nm at 500 h to 15.5 nm at 720 h and 10.2 nm at 1000 h, as shown in Table 5. Thus, although the average crystal-plane spacing remains nearly unchanged, prolonged thermal exposure results in a substantial loss of coherent crystalline-domain size and increasing structural heterogeneity. This behavior suggests possible degradation-assisted rearrangement or chemi-crystallization, in which chain scission generates shorter and more mobile segments that can reorganize and contribute to additional crystallization, as indicated by the DSC results. At the same time, degradation can introduce crystal defects, disrupt tie molecules, increase lattice disorder [5], and broaden the distribution of crystalline-domain sizes, thereby reducing the dimensions over which coherent diffraction is maintained. Consequently, continued or modest crystallization can coexist with increased XRD peak broadening and a reduction in the apparent coherent-domain size. Together with DSC analysis, the XRD results therefore suggest that the dominant effect changes from crystal refinement and packing during the early annealing stage to limited additional crystallization accompanied by the formation of smaller and/or less coherent crystalline domains during prolonged aging.

#### 3.3.4. Thermogravimetric Analysis (TGA) and Thermal Stability

To evaluate the thermal stability of PFA during aging, the temperatures corresponding to 5% and 30% mass loss, $T_{5\%}$ and $T_{30\%}$, respectively, together with the maximum decomposition temperature ($T_{max}$), were determined and are summarized in Table 6. Here, $T_{max}$ corresponds to the temperature of the maximum mass loss rate obtained from the derivative weight (DTG) peak. The thermal heat resistance index (THRI) was additionally calculated to analyze the thermal stability of PFA during the course of aging as [5,30]:

(13) $$THRI=0.49\left[T_{5\%}+0.6\left(T_{30\%}-T_{5\%}\right)\right]$$

**Figure 8:**
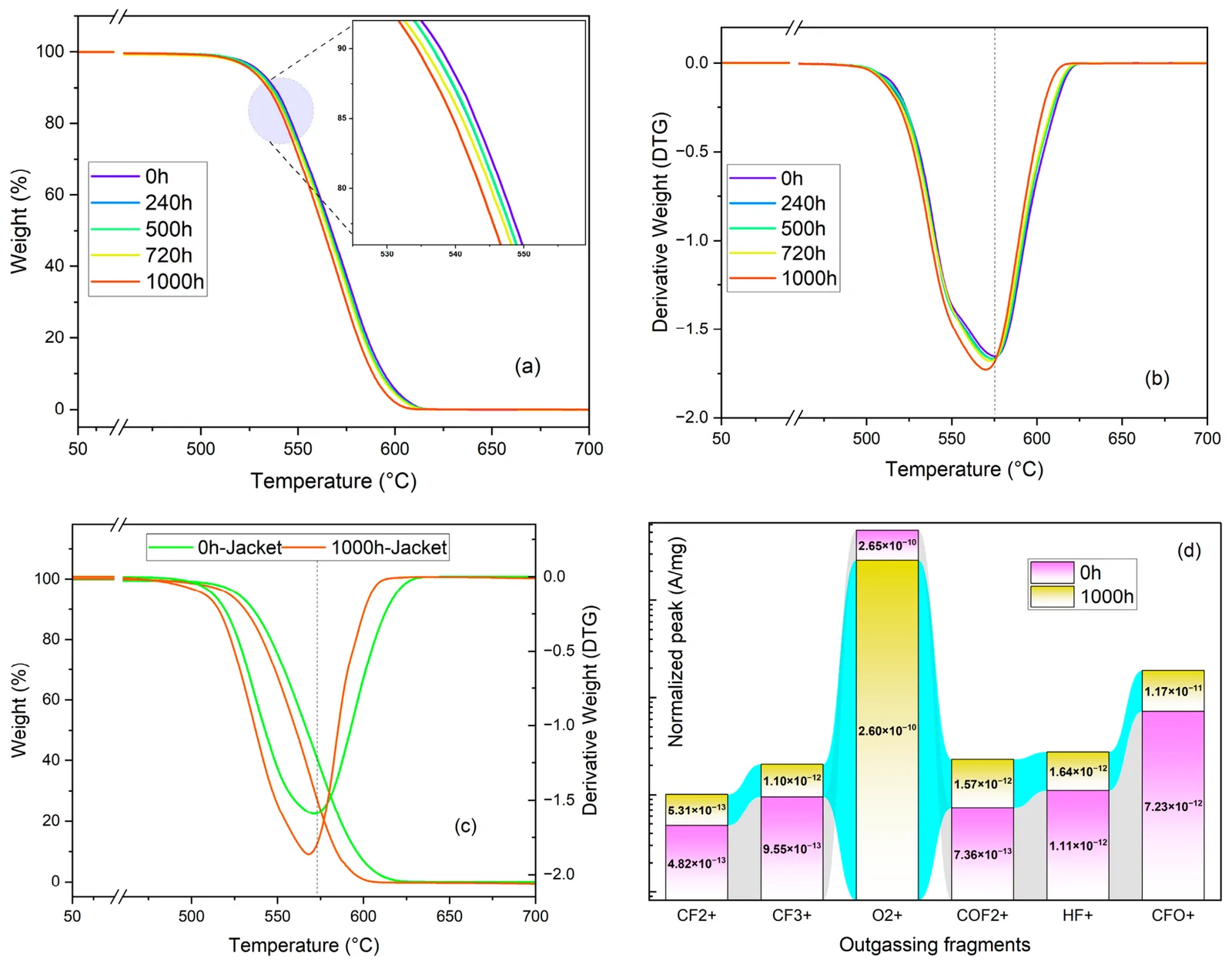
TGA, DTG and TG-MS results of PFA during aging: (**a**) TGA weight (%) of PFA insulation. (**b**) DTG of PFA insulation. (**c**) TGA weight (%) and DTG of PFA jacket. (**d**) Comparison of normalized TG–MS peak intensities of characteristic evolved-gas fragments from unaged (0 h) and thermally aged (1000 h) PFA insulation.

In general, polymers with higher thermal heat resistance and longer molecular chains tend to exhibit higher decomposition temperatures, whereas chain scission and the formation of lower-molecular-weight species reduce thermal stability and promote earlier decomposition [5,8]. As shown by the TGA curves in Figure 8a, PFA remained highly thermally stable, with negligible mass loss up to approximately 480 °C. All samples exhibited predominantly one-step decomposition, characterized by rapid mass loss above approximately 520 °C and essentially complete decomposition at higher temperatures with negligible residual mass. The unaged PFA exhibited $T_{5\%}$, $T_{30\%}$, and $T_{max}$ values of 529.0, 554.0, and 574.9 °C, respectively, confirming the high intrinsic thermal stability of the fluoropolymer. During the initial aging stage up to approximately 500 h, only minor changes in the characteristic decomposition temperatures were observed. $T_{5\%}$ decreased slightly from 529.0 to 528.3 °C, $T_{30\%}$ from 554.0 to 553.4 °C, and $T_{max}$ from 574.9 to 574.2 °C, while the THRI remained nearly unchanged, decreasing from 266.5 to 266.2 °C, as evident from Table 6. This limited variation indicates that the bulk thermal-decomposition resistance was largely preserved during the early aging period. Together with the pronounced increase in crystallinity observed by DSC and observations from FTIR analysis, this behavior supports an annealing-dominated stage, in which structural rearrangement and crystal perfection occur in PFA without substantial deterioration of the polymer backbone. Beyond approximately 500 h, the decrease in decomposition temperatures became more evident. After 1000 h, $T_{5\%}$, $T_{30\%}$, and $T_{max}$ decreased to 525.8, 550.7, and 570.4 °C, respectively, while THRI decreased to 264.9 °C. The earlier onset of thermal decomposition in the highly aged PFA samples suggests the progressive formation of thermally less stable molecular populations, which is consistent with chain scission and the generation of lower-molecular-weight segments during prolonged thermal exposure [5]. Consistently, the DTG peak in Figure 8b shifts toward lower temperature with aging, showing that the maximum rate of decomposition occurs faster and earlier in the aged PFA. This progressive reduction in decomposition resistance, despite the slight continued increase in crystallinity and lamellar thickness observed by DSC, supports a transition from predominantly physical annealing to possible degradation-assisted chemi-crystallization at prolonged aging times.

The PFA jacket exhibited an even greater reduction in thermal stability after a nominal elapsed duration of 1000 h, with $T_{5\%}$, $T_{30\%}$, and $T_{max}$ decreasing to 521.9, 548.5, and 568.5 °C, respectively, and THRI decreasing to 263.6 °C, as shown in Figure 8c and Table 6. Compared with the corresponding primary insulation, these lower decomposition temperatures indicate a somewhat greater susceptibility of the jacket to prolonged thermal degradation. When considered together with its greater increase in crystallinity, the results suggest a comparatively stronger degradation-assisted structural evolution in the jacket.

#### 3.3.5. TG–MS Evolved-Gas Analysis

To further investigate the chemical evolution suggested by FTIR, the volatile products generated during thermal decomposition of unaged and 1000 h aged PFA were analyzed by TG–MS. The monitored ions included CF_2_^+^ (m/z 50), HF^+^ (m/z 20), COF_2_^+^ (m/z 66), CFO^+^ (m/z 47), and CF_3_^+^ (m/z 69). After normalization to the initial sample mass, the aged PFA exhibited systematically higher ion-current responses than the unaged material, although the magnitude of the increase differed substantially among the detected species (Figure 8d and Table 7). The CF_2_^+^ peak increased from $4.8\times 10^{-13}$ to $5.3\times 10^{-13}$ A mg^−1^, corresponding to an increase of approximately 10.4%, while CF_3_^+^ increased from $9.5\times 10^{-13}$ to $1.1\times 10^{-12}$ A mg^−1^, corresponding to approximately 15.7%. A plausible degradation mechanism involves scission of the polymer backbone, generating CF_2_-containing radical species, which may subsequently undergo fluorination or recombination reactions to form CF_3_ molecules [31]. These relatively modest changes suggest that the fluorocarbon framework remains largely preserved after 1000 h of aging, which is in good agreement with the FTIR results showing no severe degradation of the dominant PFA backbone bands. In contrast, considerably larger increases were observed for the oxygen-containing and fluorine-containing degradation products. The HF^+^ signal increased by approximately 47.7%, COF_2_^+^ by approximately 112%, and CFO^+^ by approximately 61.8%. This preferential enhancement of HF-, COF_2_-, and CFO-related signals indicates that prolonged aging increases the susceptibility of specific fluorinated and oxygen-containing environments to subsequent thermal decomposition. This behavior is consistent with reported degradation mechanisms of PFA, in which limited chain scission and cleavage involving ether-containing side-chain structures can generate HF-, CF_3_- and CFO-containing species through radical fragmentation and fluorine-transfer reactions [32]. Previous structural studies of PFA have likewise shown that chain scission can produce CF_3_, COF and related end-group structures, supporting the assignment of these species as indicators of localized chemical degradation [33]. Under elevated temperatures, particularly during thermal degradation, PFA can also release fluorinated decomposition products including HF and carbonyl fluoride (COF_2_) [5,34]. Accordingly, the present results may be interpreted as evidence that prolonged thermal aging induces limited chain scission and cleavage within the ether-containing side-chain environment, increasing the tendency to generate CFO, COF_2_ and HF, containing fluorinated volatile products during subsequent heating.

A slight shift in the evolved-gas maxima toward lower temperature was also observed after aging. The CF_2_^+^ peak shifted from 567 to 559 °C, HF^+^ from 571 to 563 °C, and both COF_2_^+^ and CFO^+^ from 563 to 559 °C, while CF_3_^+^ remained centered at approximately 563 °C, as shown in Table 7. This modest 4–8 °C reduction in the evolution temperature indicates that prolonged aging slightly lowers the thermal stability of the more susceptible chemical environments, causing fragmentation and volatile release to occur more readily during the subsequent TG–MS scan. These TG–MS results correlate well with the FTIR observations. During the earlier aging stage, the main changes were concentrated in the 720–800 cm^−1^ region and were attributed primarily to conformational rearrangement and increased molecular ordering. At prolonged aging times, stronger changes were observed around the ether-associated C–O–C band near 995 cm^−1^. The enhanced evolution of CFO^+^ and COF_2_^+^ from the 1000 h sample therefore provides complementary evidence for the increasing involvement of oxygen-containing structural environments during prolonged aging. At the same time, the relatively small changes in CF_2_^+^ and CF_3_^+^ remain consistent with the preservation of the principal fluorocarbon framework observed by FTIR. Collectively, the TG–MS results indicate that prolonged aging introduces limited degradation-assisted chemical evolution involving chain/ether-region cleavage and enhanced formation of volatile fluorinated products.

### 3.4. Morphological and Surface Evolution

#### 3.4.1. Surface and Bulk Morphology

**Figure 9:**
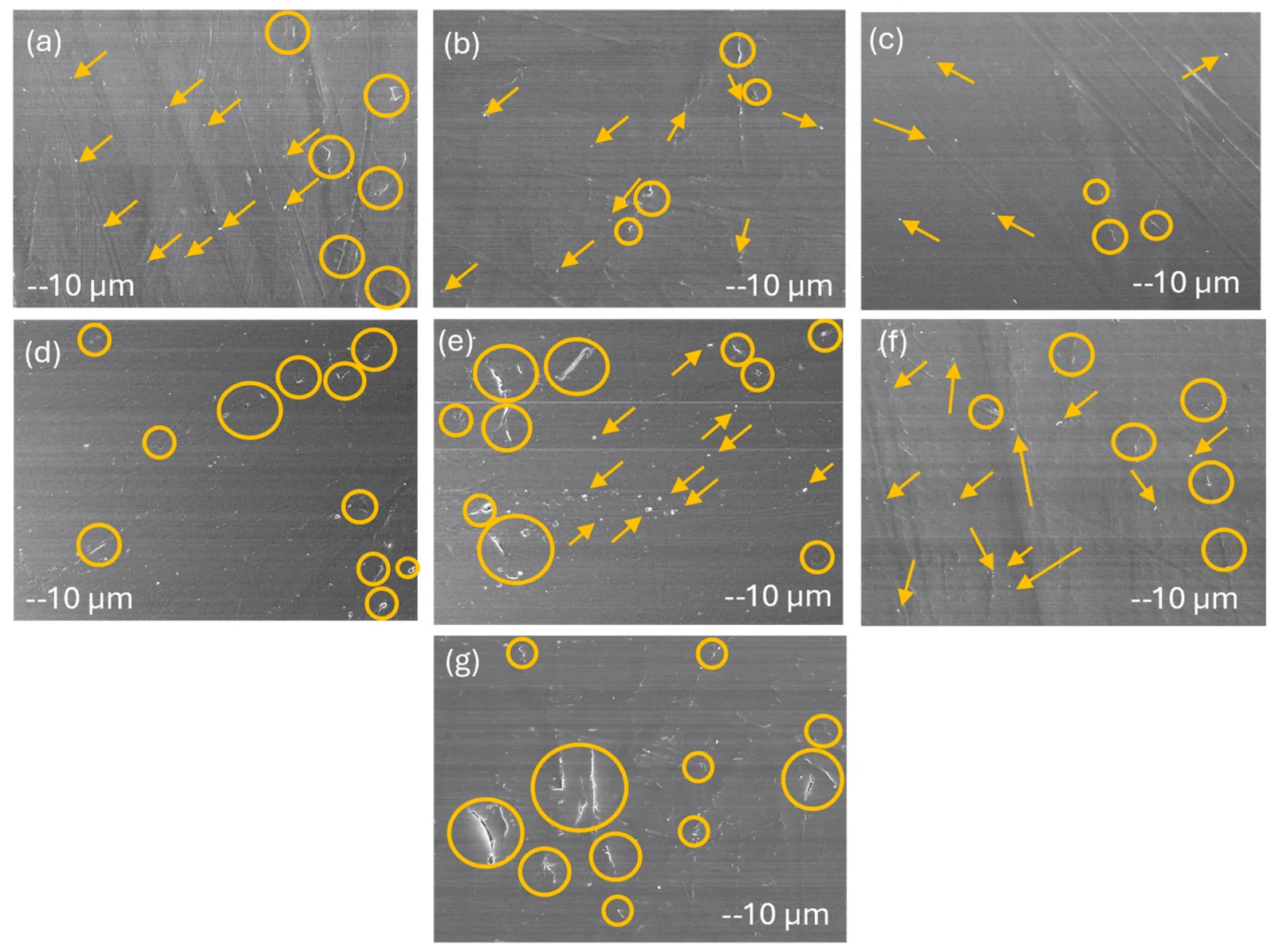
SEM surface morphological evolution of PFA: (**a**–**e**) PFA insulation (0–1000 h). (**f**) PFA jacket (0 h). (**g**) PFA jacket (1000 h).

**Figure 10:**
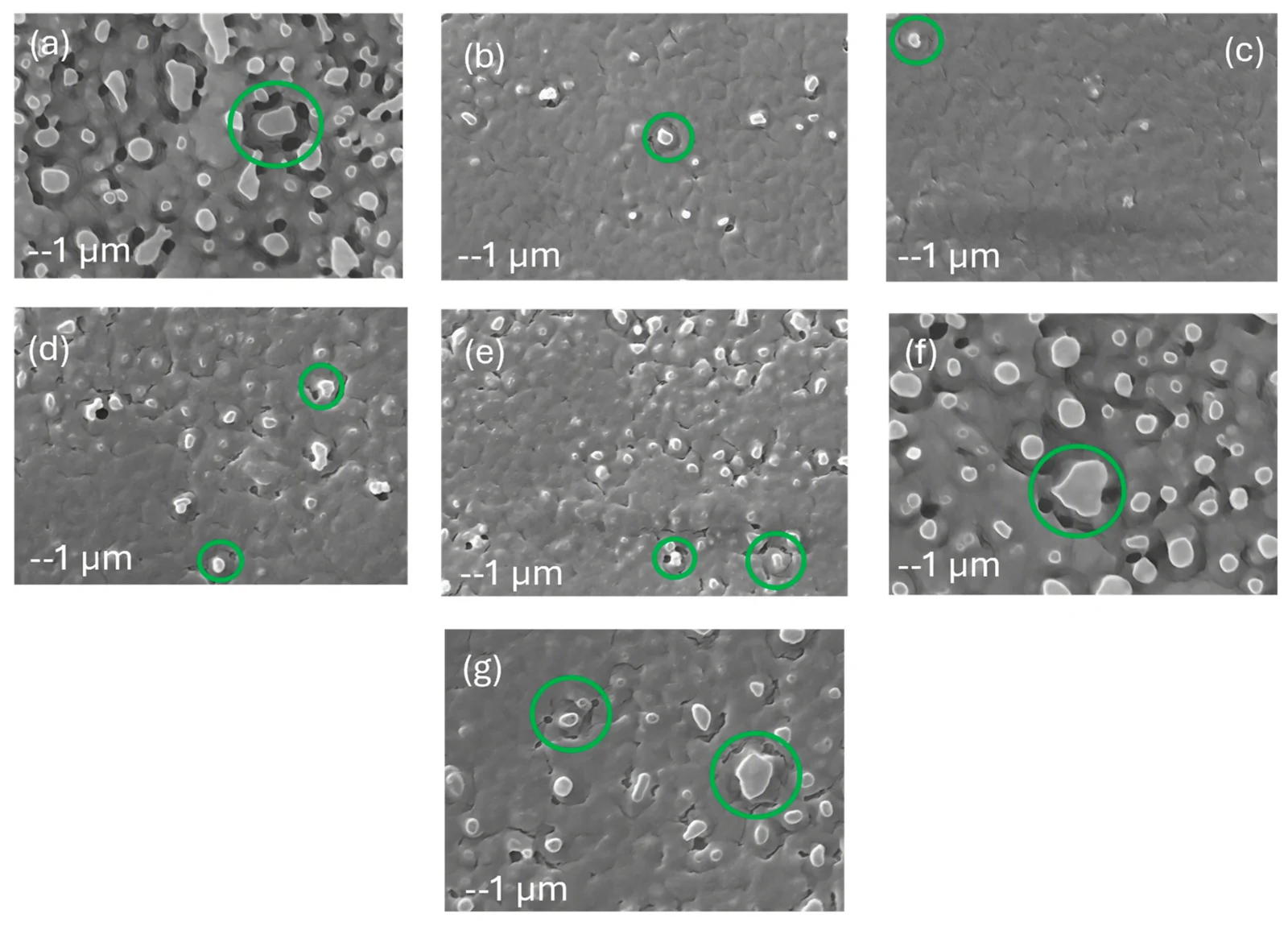
SEM bulk morphological evolution of PFA: (**a**–**e**) PFA insulation (0–1000 h). (**f**) PFA jacket (0 h). (**g**) PFA jacket (1000 h).

Figure 9a–g shows the surface morphology of PFA, while Figure 10a–g presents the corresponding bulk morphology. The unaged PFA surface, Figure 9a, is relatively smooth and continuous, with shallow striations, minor protrusions, and isolated depressions that are likely associated with extrusion, specimen preparation, or handling. In contrast, the unaged bulk morphology in Figure 10a is comparatively heterogeneous, containing numerous nodular or crystal-associated domains, distinct boundary-like contrasts, and void-like depressions. These features indicate a relatively open and non-uniform initial microstructure. After 240 h of nominal elapsed duration, the surface becomes denser and comparatively more homogeneous, with fewer pronounced protrusions and irregularities, as shown in Figure 9b. A corresponding reduction in large nodular features, boundary-like discontinuities, and void-like depressions is observed in the bulk morphology in Figure 10b. After 500 h, the surface in Figure 9c exhibits the smoothest and most continuous morphology among the investigated insulation specimens, with only a small number of isolated imperfections. The corresponding bulk morphology in Figure 10c is also more compact and homogeneous, with fewer visible boundary defects and void-like regions than the unaged material. The morphological consolidation observed between 240 and 500 h is consistent with the physical-annealing-dominated behavior established by the preceding FTIR and DSC analyses. At the sub-melting aging temperature, increased segmental mobility may facilitate residual stress relaxation, local chain sliding, molecular rearrangement, and tighter packing within the semicrystalline structure [11]. These processes reduce the prominence of boundary-like discontinuities and produce a denser and more uniform morphology. Similar behavior has been reported for sub-melting annealing of PFA, where molecular-chain rearrangement and crystal perfection resulted in increased lamellar thickness and reduced boundary defects [11]. Although individual lamellae cannot be resolved sufficiently to quantify interlamellar spacing in the present SEM images, the reduced boundary-like discontinuities and void-like features provide complementary morphological evidence of this internal reorganization. The 500 h PFA specimen consequently exhibits the highest morphological uniformity and lowest apparent defect density.

After 720 h of nominal elapsed duration, the surface remains largely continuous but becomes comparatively less uniform, as shown in Figure 9d. Isolated pits, shallow grooves, irregular depressions, and short fissure-like features become more evident. The corresponding bulk morphology in Figure 10d also contains a greater number of small cavities, irregular boundaries, and localized separations around nodular regions. These observations indicate a transition from the earlier stage of microstructural consolidation to the onset of irreversible thermal aging damage. This transition is in good agreement with the preceding FTIR results indicating degradation-assisted molecular rearrangement and possible chemi-crystallization, together with the reduced thermal stability detected by TGA after prolonged aging [5]. At 1000 h, the PFA insulation surface in Figure 9e becomes increasingly heterogeneous and appears rougher, with a higher concentration of pits, irregular grooves, surface undulations, fine fissure-like features, and bright nodular domains. The corresponding bulk morphology in Figure 10e is also less uniform and contains numerous cavities, irregular boundary-like regions, and a more open granular or nodular structure. These alterations may arise from the internal structural adjustments associated with chain degradation, contributing to a non-uniform distribution of stress to the PFA surface. Chain scission processes can generate shorter, lower-molecular-weight chain segments with increased mobility [5]. Local rearrangement or migration of these species toward the surface may subsequently facilitate crystal nucleation and growth. A comparable mechanism involving chain scission, localized stress relaxation, migration of lower-molecular-weight species, and surface crystal formation has been reported in the literature [35]. Thus, the simultaneous appearance of crystal-associated nodular features, cavities, and fissures supports degradation-assisted rearrangement and possible chemi-crystallization [5].

The PFA jacket, after a nominal elapsed duration of 1000 h, exhibits more pronounced morphological deterioration than the corresponding insulation specimen, when compared with its unaged condition in Figure 9f. As shown in Figure 9g, the jacket surface contains wider and deeper crack-like openings, together with numerous pits, protrusions, and irregular depressions. Similarly, the bulk morphology in Figure 10g is coarser and more heterogeneous, with larger cavities, nodular domains, and localized separations. These features are consistent with a greater extent of thermal aging damage and potentially more pronounced degradation-assisted rearrangement in the jacket. Differences in formulation, processing history, or residual stress may contribute to its greater susceptibility. The larger cavities and crack-like defects may also act as local stress concentration sites and contribute to the subsequent deterioration of the jacket’s mechanical and dielectric properties.

#### 3.4.2. Surface Elemental Composition

**Figure 11:**
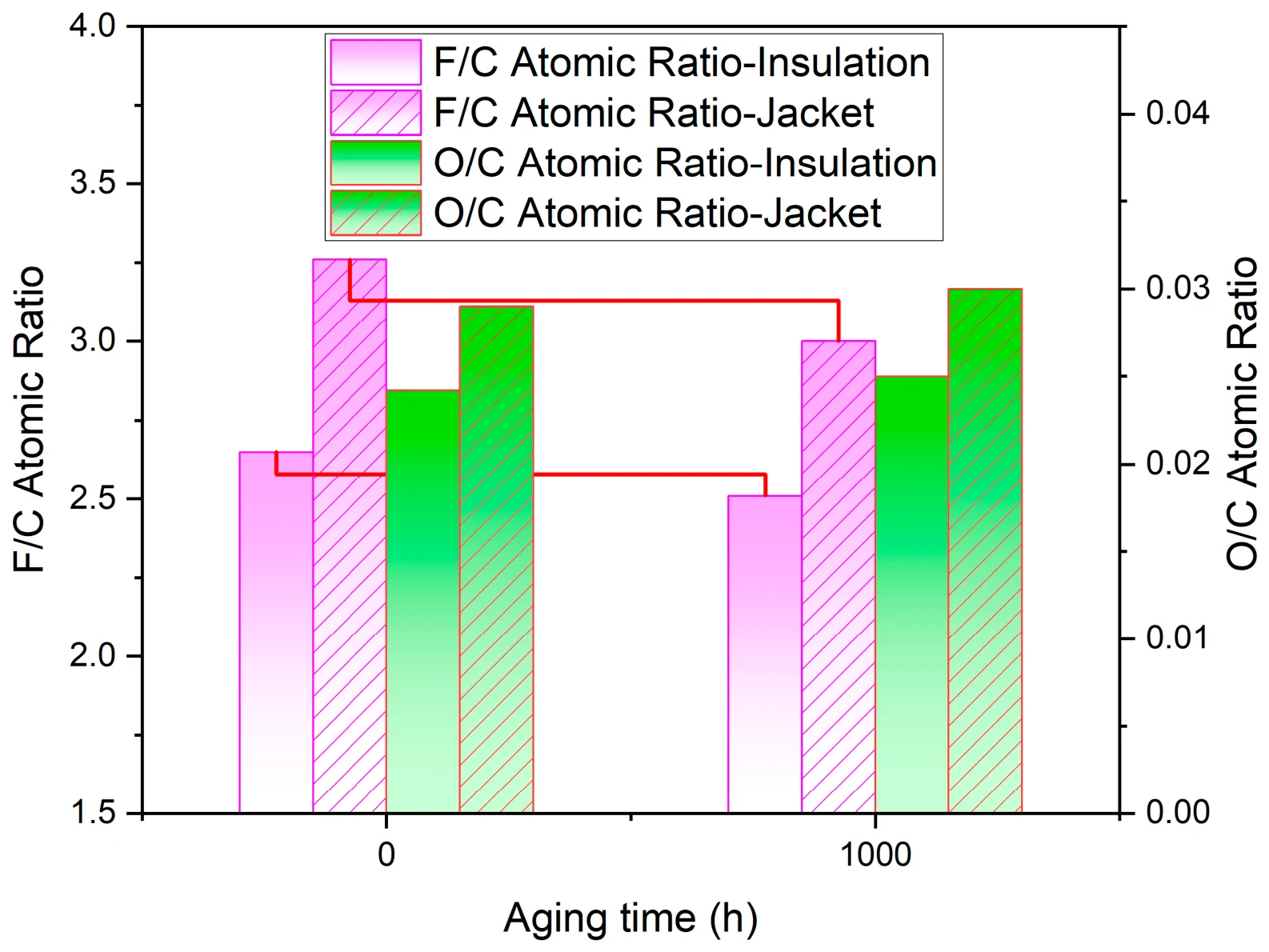
Comparison of atomic fraction ratios of PFA insulation and jacket.

EDS was employed as a semi-quantitative technique to evaluate aging-induced changes in the surface elemental composition of PFA and to complement the preceding FTIR, thermal, TG–MS, and morphological analyses. During the early aging stage, from 0 to 500 h, the fluorine content remained largely unchanged, indicating substantial retention of the fluorinated surface structure, as shown in Table 8. This behavior agrees well with the FTIR, DSC, and TGA results, which indicate that this period is predominantly governed by physical annealing and structural reorganization rather than extensive chemical degradation. After prolonged aging, a small but systematic decrease in the surface fluorine concentration was observed. The fluorine atomic fraction decreased from 72.2 at.% for the unaged PFA to 71.0 at.% after 1000 h. To evaluate the relative evolution of the surface composition, the F/C and O/C atomic ratios were calculated directly from the EDS-derived atomic fractions according to F/C = (F at.%)/(C at.%) and O/C = (O at.%)/(C at.%). The use of elemental atomic ratios derived from EDS/EDX measurements is an established comparative approach in polymer aging studies. For example, C:F ratios have been used to evaluate thermal aging-induced compositional changes in PTFE, while C:O, C:N and O/C ratios have been employed to follow surface chemical evolution in PEEK, PI and EPDM [8,36]. Similar SEM/EDS analysis has also been applied to thermally aged PFA to assess surface fluorine loss and degradation [5]. In the present study, the F/C ratio decreased progressively from 2.65 in the unaged condition to 2.51 after 1000 h, corresponding to a reduction of approximately 5.2% (Figure 11). The ratios are used here as relative EDS-derived compositional indicators, rather than as absolute stoichiometric values of PFA, because EDS quantification of light elements is semi-quantitative and can be affected by detector response, matrix effects, interaction volume, surface morphology, and the applied quantification procedure. Nevertheless, when measurements are performed under the same acquisition and analysis conditions, elemental ratios provide a useful basis for comparing relative surface-compositional changes among aged specimens, as demonstrated in previous polymer aging studies [5,8,36]. The decrease in F/C is therefore interpreted as being consistent with limited fluorine loss from the near-surface region, rather than as standalone evidence of defluorination. Importantly, this trend is independently supported by the preceding TG–MS analysis. After 1000 h, the normalized peak intensities of HF^+^, COF_2_^+^ and CFO^+^ increased by approximately 47.7%, 112%, and 61.8%, respectively, compared with the unaged PFA. The enhanced evolution of these fluorine-containing volatile species demonstrates that the aged material has an increased tendency to release fluorinated decomposition products during subsequent thermal degradation. Together with the reduction in the EDS-derived F/C ratio, these results support the occurrence of limited defluorination and localized chemical modification during prolonged thermal aging. In contrast, the O/C ratio changed only marginally from 0.024 at 0 h to 0.025 at 1000 h. Considering the semi-quantitative nature of EDS for light elements, this small variation does not provide evidence for substantial accumulation of oxygen-containing species at the PFA surface. The weak oxygen signal may additionally be influenced by adventitious surface contamination, sample preparation, surface morphology, or instrumental uncertainty [36]. The nearly unchanged O/C ratio, together with the absence of pronounced new oxidation-related FTIR bands, therefore indicates that persistent oxidative modification of the PFA surface remained comparatively limited under the applied aging conditions.

The surface-compositional evolution is consistent with the chemical mechanism inferred from the combined FTIR and TG–MS analyses. Thermal degradation in PFA is proposed to initiate preferentially at vulnerable bonds near the ether-containing side-chain region, particularly the –O–CF_2_– linkage, where cleavage of C–O–C and adjacent C–C bonds can generate radical species and shorter fluorinated chain fragments [5]. This interpretation is supported by the gradual weakening of the ether-related C–O–C band observed by FTIR and by the enhanced evolution of CFO^+^ and COF_2_^+^ detected by TG–MS. At the same time, the comparatively modest changes in the CF_2_^+^ and CF_3_^+^ TG–MS signals and the preservation of the principal fluorocarbon FTIR bands indicate that the main PFA fluorocarbon framework remains largely intact and that severe bulk degradation has not occurred. The compositional changes also correlate with the morphological evolution observed by SEM. The increasing occurrence of pits, cavities, and fissure-like defects at 720–1000 h coincides with the gradual decrease in F/C and the reduction in thermal stability observed by TGA.

The PFA jacket exhibited a greater relative decrease in fluorine content than the primary insulation, as illustrated in Figure 11 and Table 9. Its F/C ratio decreased from 3.26 in the unaged condition to 3.00 after 1000 h, representing a reduction of approximately 8.0%, compared with approximately 5.2% for the insulation. This greater reduction indicates more pronounced surface defluorination of the jacket and is consistent with its more extensive cracking, cavities, and morphological heterogeneity observed by SEM. Direct exposure of the outer PFA jacket to the high-temperature environment, together with possible differences in formulation, processing history, and initial morphology, may contribute to its greater susceptibility. Overall, thermal aging for a nominal elapsed duration of 1000 h produces relatively modest changes in the elemental composition of PFA, characterized principally by limited surface defluorination with no clear evidence of substantial oxidation.

#### 3.4.3. Static Contact Angle Analysis

**Figure 12:**
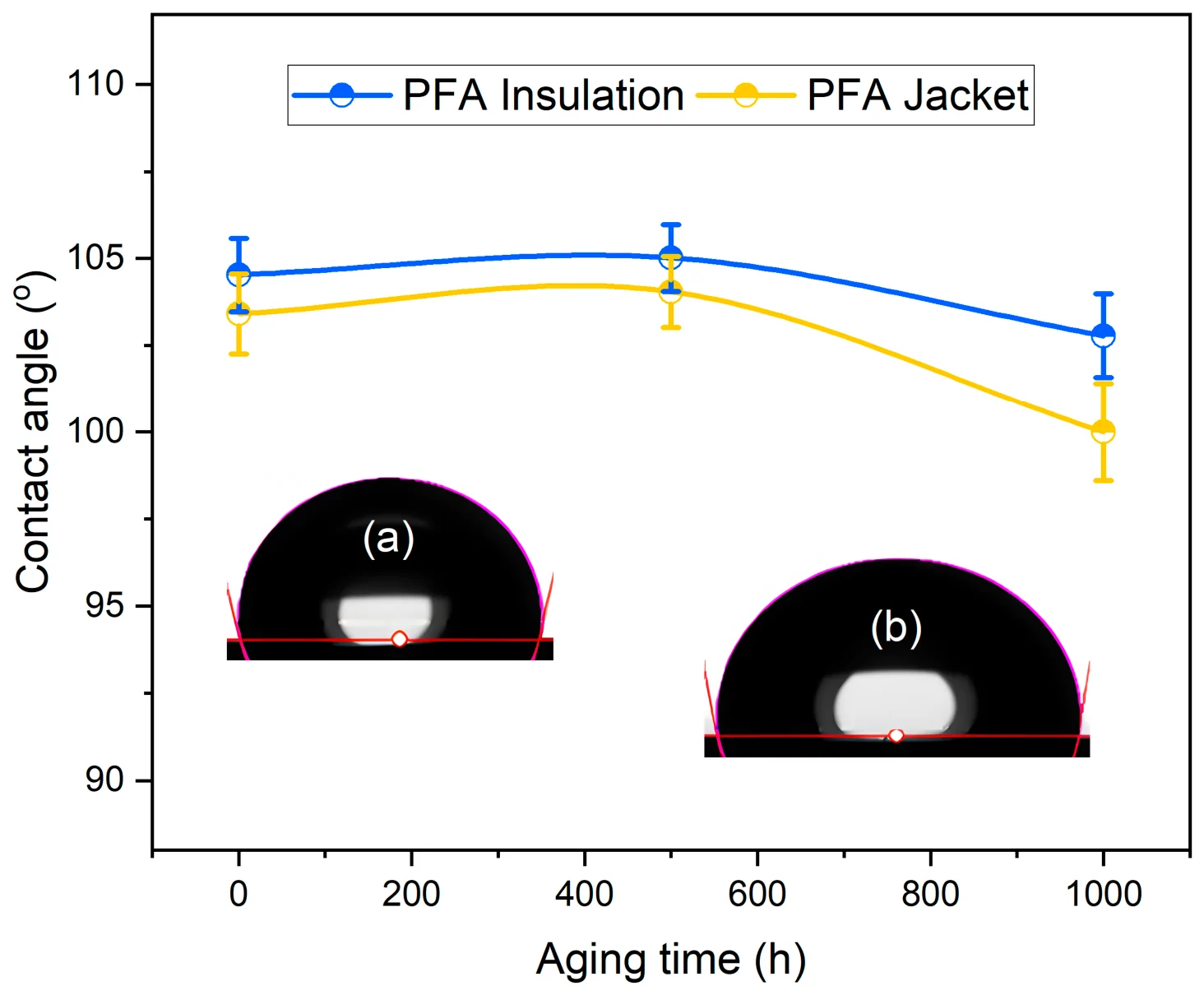
Surface wettability of PFA insulation and jacket during aging.

The evolution of surface wettability was evaluated using static water contact angle measurements, as shown in Figure 12. The unaged PFA insulation exhibited a contact angle of 104.52°, confirming the hydrophobic nature of the surface. After 500 h, the contact angle increased slightly to 105.01°, whereas prolonged exposure to 1000 h reduced it to 102.78°. The contact angle response therefore exhibits two stages: a minor increase during the first 500 h, followed by a decrease between 500 and 1000 h. The initial increase is consistent with the early-stage physical annealing and structural ordering identified by FTIR and DSC and with the smoother, more compact morphology observed by SEM. In contrast, the reduction after 1000 h corresponds to the later-stage degradation indicated by FTIR, TGA and TG-MS and to the increased surface heterogeneity, fissure-like defects, and limited defluorination observed by SEM and EDS. According to the Wenzel model, surface roughness amplifies the intrinsic wetting behavior of a material, provided that the liquid completely penetrates the surface asperities [37,38]. For chemically unchanged hydrophobic PFA, increased roughness alone would generally increase the apparent contact angle. Consequently, the measured reduction after prolonged aging is attributed primarily to aging-induced changes in surface chemistry, particularly limited defluorination and the development of more heterogeneous and potentially more wettable defect regions. The cracks, pits, and cavities may additionally facilitate local droplet spreading, liquid penetration into surface irregularities, and contact-line pinning, thereby contributing to the lower apparent contact angle.

The PFA jacket exhibited a larger relative reduction in contact angle after 1000 h of approximately 3.2%, compared with approximately 1.7% for the primary insulation. This behavior is consistent with the greater surface defluorination, cracking, and morphological heterogeneity observed for the jacket by EDS and SEM. This also supports the observation previously discussed in DSC analysis in which the PFA jacket indicated a somewhat greater susceptibility to the possible degradation-assisted chemi-crystallization. In the jacket specimens, the water droplet changed from a relatively spherical configuration in the unaged condition, Figure 12(a), to a more spread configuration after 1000 h, Figure 12(b), indicating increased water–surface interaction. Nevertheless, the contact angle remained above 90°, confirming that the aged PFA surface retained an overall hydrophobic character. The larger decrease in contact angle observed for the jacket, together with its pronounced susceptibility towards defluorination and surface damage, indicates deterioration of its surface barrier condition. Although PFA generally exhibits high chemical stability, hydrophobicity, and low moisture uptake, its resistance to moisture has been reported to be somewhat lower than that of PTFE [39]. The cracks, pits, and chemically heterogeneous regions formed during prolonged aging may therefore provide preferential pathways for the ingress of moisture, chemicals, and other environmental contaminants. Such ingress could increase the exposure of the underlying insulation and conductor to external species and potentially affect the long-term mechanical, dielectric, and protective performance of the cable. Overall, the contact angle results complement the preceding spectroscopic, thermal, elemental, and morphological analyses of PFA.

### 3.5. Thermomechanical and Mechanical Behavior

#### 3.5.1. Dynamic Mechanical Response

**Figure 13:**
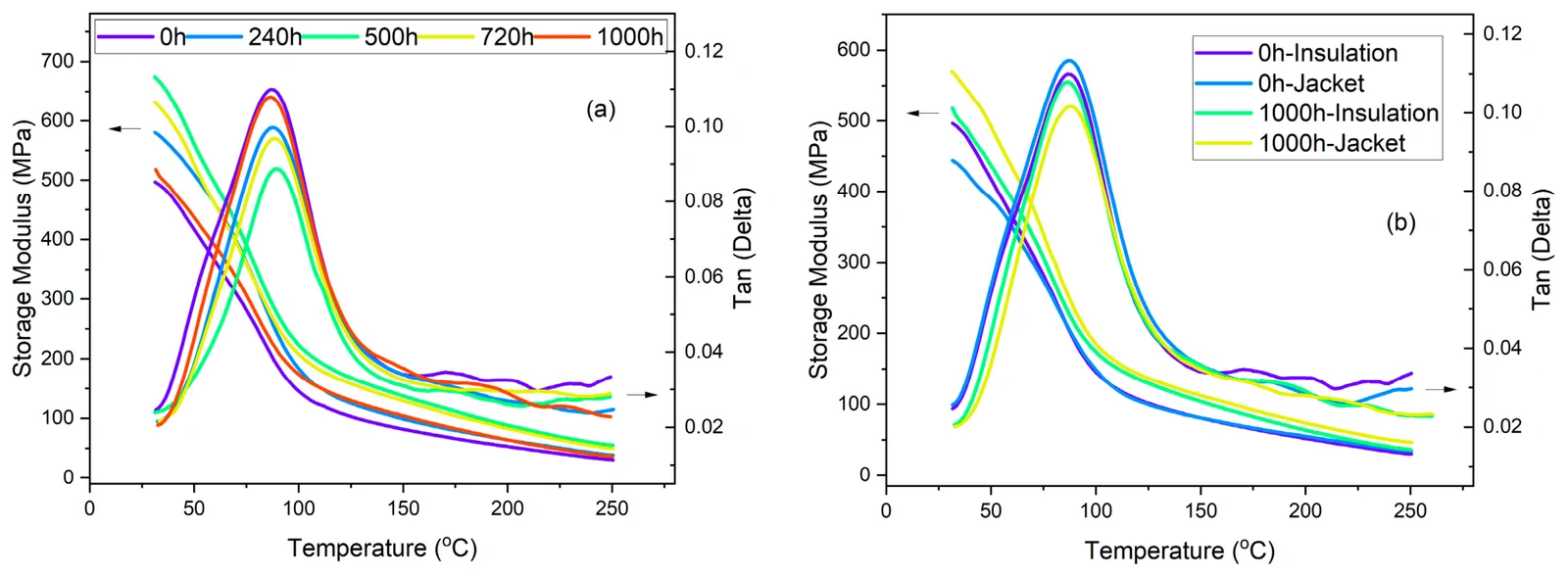
DMA response of PFA: (**a**) Storage modulus and Tan (delta) of PFA insulation. (**b**) Comparison of storage modulus and Tan (delta) of PFA insulation and jacket at different aging times.

The temperature-dependent storage modulus ($E^{\prime }$) and loss factor (tan δ) responses of the PFA insulation and jacket are shown in Figure 13, while the characteristic DMA parameters are summarized in Table 10. For both PFA layers, $E^{\prime }$ decreases with increasing temperature, with a pronounced reduction in the vicinity of 70–100 °C corresponding to the glass-transition region. The tan δ peak observed at approximately 86–90 °C is assigned to the α-relaxation associated with cooperative segmental motion of the amorphous chains [4,5,40]. During the initial aging stage up to a nominal elapsed duration of 500 h, the storage modulus increases markedly. For the PFA insulation, $E^{\prime }$ increases from 208.7 MPa in the unaged state to 276.7 MPa at 500 h, corresponding to an increase of approximately 32.6%. A stronger increase is observed for the PFA jacket, from 208.3 to 300.1 MPa, corresponding to approximately 44.1%. This greater stiffening of the jacket is consistent with its larger initial increase in crystallinity observed by DSC. Concurrently, $T_{g}$ shifts from 86.5 to 89.6 °C for the insulation and from 86.5 to 90.0 °C for the jacket. The tan δ peak also decreases in intensity and becomes sharper. These changes indicate reduced damping, restricted segmental mobility, and a narrower distribution of relaxation environments [8,41]. The combined increase in $E^{\prime }$ and $T_{g}$, together with the sharper and weaker α-relaxation, supports an annealing-dominated structural reorganization, involving improved chain packing, crystalline development or perfection, and increased constraint of the remaining amorphous chains [11].

Beyond 500 h, the storage modulus progressively decreases, as illustrated in Figure 13. For the PFA insulation, E′ falls from 276.7 MPa at 500 h to 230.5 MPa at 1000 h, while the jacket decreases from 300.1 to 250.2 MPa, as shown in Table 10. Nevertheless, the 1000 h modulus remains approximately 10.4% above the unaged PFA value for the insulation and 20.1% above that of the unaged PFA jacket, as illustrated in Figure 13b. Thus, prolonged aging does not completely eliminate the stiffening developed during the initial aging stage; rather, it partially counteracts the annealing-induced enhancement in rigidity of PFA. The decline of $E^{\prime }$ in PFA layers is consistent with the increasing contribution of chain scission in the amorphous regions, increased free volume of the residual amorphous phase, loss of molecular connectivity, and microstructural damage [5]. These changes provide indirect evidence of a reduction in effective molecular weight associated with thermal degradation, as reported for fluoropolymers [5,8,42]. Chain cleavage generates shorter molecular segments and reduces effective entanglement density, thereby weakening the load-bearing contribution of the amorphous network and lowering $E^{\prime }$ in PFA [8]. At the same time, some shorter chain segments may reorganize into crystalline regions through degradation-assisted crystallization or chemi-crystallization; therefore, retained or increased crystallinity can coexist with a reduction in dynamic stiffness. The evolution of the α-relaxation in PFA further supports this transition. After reaching maximum values at 500 h, $T_{g}$ decreases to 86.6 °C for the PFA insulation and 87.3 °C for the jacket at 1000 h. In contrast to the early aging stage, the tan δ peak becomes broader and more intense during prolonged aging as illustrated in Figure 13a. The shift toward lower temperature and increase in damping indicate enhanced segmental mobility, while peak broadening suggests a wider distribution of relaxation times and increasing microstructural heterogeneity in PFA. This behavior is consistent with the coexistence of crystalline regions, constrained interfacial chains, and shorter degradation-affected amorphous segments. The PFA jacket consistently exhibits a stronger aging response than the primary insulation. At 500, 720, and 1000 h, its storage modulus is approximately 8.5%, 10.5%, and 8.5% higher, respectively, than that of the corresponding insulation. These observations, together with the DSC results, indicate that structural rearrangement and crystallinity development occur more extensively in the PFA jacket during the initial aging stage, resulting in a greater increase and greater retention of stiffness.

#### 3.5.2. Tensile Properties

**Figure 14:**
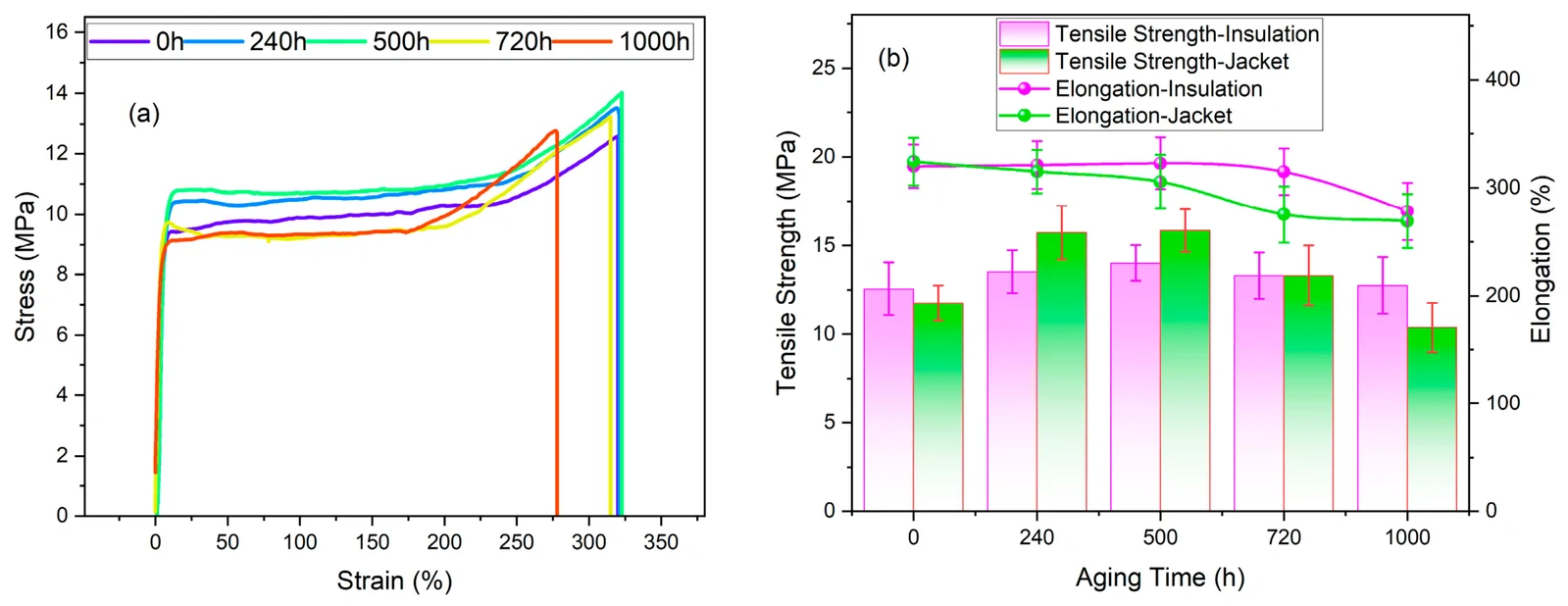
Tensile response of PFA: (**a**) Stress–strain curves of PFA insulation. (**b**) Comparison of tensile strength and elongation of PFA insulation and jacket at different aging times.

The stress–strain curves of PFA insulation at various stages of the aging process are shown in Figure 14a, and the corresponding tensile parameters are listed in Table 11. During the initial aging period up to a nominal elapsed duration of 500 h, both PFA layers show increased stiffness and strength, as evident from the results in Table 11. For the PFA insulation, Young’s modulus increases from 252 to 336 MPa, while the tensile strength increases from 12.56 to 14.02 MPa, corresponding to increases of approximately 33.3% and 11.6%, respectively. The yield stress also increases from 9.5 to 10.7 MPa, as highlighted in Table 11. A stronger response is observed for the jacket, for which Young’s modulus increases from 249 to 345 MPa and tensile strength from 11.76 to 15.85 MPa, corresponding to increases of approximately 38.6% and 34.8%, respectively. The jacket yield stress similarly increases from 10.0 to 12.5 MPa. The greater early-stage strengthening of the jacket agrees with its larger increase in crystallinity observed by DSC and its greater storage modulus enhancement measured by DMA. The improvement in mechanical properties during this period is attributed predominantly to annealing-induced structural reorganization, including improved molecular packing, crystalline development and lamellar perfection [11]. In semicrystalline polymers, the elastic response is governed not only by the crystalline fraction but also by lamellar organization, tie-molecule connectivity, and mobility of the interlamellar amorphous phase. Increased crystalline constraint therefore reduces chain mobility and improves stress transfer between crystalline and amorphous regions, resulting in increased Young’s modulus, yield stress, and tensile strength [11,21,24,43,44]. This interpretation agrees well with the DMA results, where the storage modulus and $T_{g}$ increase up to approximately 500 h, accompanied by a lower and sharper tan δ α-relaxation peak, indicating reduced segmental mobility and increased structural constraint. The elongation-at-break response further reveals an important difference between the insulation and jacket during the early aging stage, as illustrated in Figure 14b. The PFA insulation retains essentially unchanged ductility, increasing slightly from 319.9% to 322.7% at 500 h. This suggests that crystalline reorganization and improved load transfer occur without substantially restricting the deformability of the remaining amorphous phase. In contrast, the PFA jacket elongation decreases from 324.1% to 305.5% (see Figure 14b), despite its pronounced increase in modulus and tensile strength. This behavior indicates that the stronger crystalline development of the jacket imposes greater restriction on amorphous-chain mobility, producing an earlier tendency toward reduced ductility. The result is therefore consistent with the stronger crystallinity evolution observed by DSC and the greater restriction of molecular mobility indicated by DMA for the jacket.

Beyond approximately 500 h, the mechanical response begins to deteriorate. For the PFA insulation, tensile strength decreases from 14.02 MPa at 500 h to 13.31 MPa at 720 h and 12.75 MPa at 1000 h (see Figure 14b), while Young’s modulus decreases from its maximum of 336 MPa to 309 MPa at 1000 h. The PFA jacket exhibits a more pronounced deterioration, as shown in Figure 14b: tensile strength decreases from 15.85 MPa at 500 h to 13.30 MPa at 720 h and 10.38 MPa at 1000 h, while Young’s modulus decreases from 345 to 315 MPa, as evident from Table 11. The yield stress follows a similar trend, decreasing to 9.1 MPa for the insulation and 9.9 MPa for the jacket after 1000 h. These reductions indicate that the strengthening achieved during the early annealing stage is progressively counteracted by incipient chain scission, reduced molecular connectivity, degradation of tie molecules, and development of microstructural defects in PFA. Chain cleavage decreases effective molecular weight and entanglement density, thereby reducing the efficiency of stress transfer between neighboring crystalline domains through the amorphous phase [5,45]. This interpretation agrees well with the later-stage DMA response, where the storage modulus decreases and the tan δ relaxation becomes broader and more intense, indicating enhanced mobility and increasing heterogeneity of the residual amorphous phase. It also agrees with the DSC results, indicating possible degradation-assisted crystallization or chemi-crystallization at prolonged aging times. Shorter chains generated by limited chain scission may reorganize into additional or more ordered crystalline regions; however, this increase in crystalline organization occurs concurrently with deterioration of the amorphous tie-chain network. Thus, retained or enhanced crystallinity does not necessarily translate into continued improvement in tensile performance. The loss of ductility becomes particularly evident at prolonged aging times. The PFA insulation elongation decreases from 322.7% at 500 h to 314.8% at 720 h and 278.0% at 1000 h (see Figure 14b), whereas the jacket decreases more strongly from 305.5% to 275.2% and finally 269.1%. Relative to the unaged state, the elongation at break decreases by approximately 13.1% for the insulation and 17.0% for the jacket after 1000 h. This progressive reduction in deformability can be attributed to the combined effects of chain scission, loss of effective entanglements and tie molecules, increased crystalline constraint, and accumulation of structural defects, which promote local stress concentration and premature failure during deformation [5]. The effect is more pronounced in the jacket, consistent with its stronger initial crystallization and subsequent degradation response.

Overall, the tensile results corroborate the thermal aging mechanism identified from DSC and DMA. Notably, Young’s modulus remains above the unaged value even after 1000 h, whereas tensile strength and elongation, particularly for the jacket, decline substantially. This combination of retained stiffness with decreasing strength and deformability indicates a transition toward a stiffer but progressively less ductile and more damage-sensitive microstructure.

#### 3.5.3. 3PB Response of the PFA Cable

**Figure 15:**
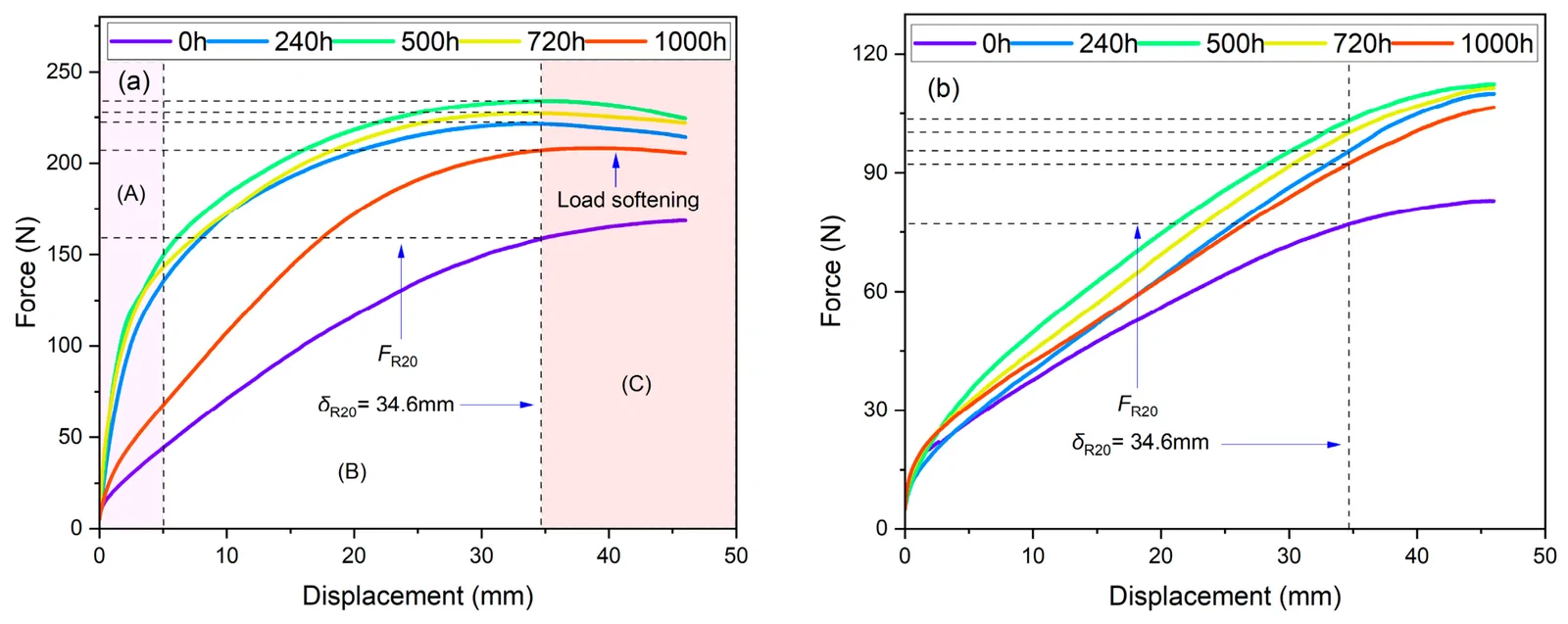
3PB response of PFA: (**a**) Force–displacement curves of PFA full cable at different aging times. (**b**) Force–displacement curves of PFA cable (jacket-removed configuration).

Figure 15a shows the force–displacement response of the complete PFA cable after thermal aging, while Figure 15b presents the corresponding response after removal of the outer PFA jacket. The bending response of the complete PFA cable can be divided phenomenologically into three regimes, as shown in Figure 15a. Region A represents the initial stiffness-dominated response used for determination of the apparent bending rigidity and apparent flexural modulus [46]. This is followed by a progressive nonlinear bending regime (Region B), in which the incremental force required for further displacement gradually decreases. Region C corresponds to deformation beyond the recommended minimum bending radius. Because the specimen is a multilayer cable and undergoes large deformation, this region is better described as a beyond-recommended-bend-radius or over-bending regime.

Thermal aging produced a pronounced non-monotonic change in the bending resistance of the complete PFA cable. The apparent bending rigidity increased from 1.39 × 10^6^ N·mm^2^ for the unaged cable to 2.96 × 10^6^ N·mm^2^ after 240 h and reached a maximum of 3.46 × 10^6^ N·mm^2^ after 500 h, as shown in Table 12. This corresponds to an increase of approximately 149% relative to the unaged state. Thereafter, ${EI}_{app}$ decreased, falling to 3.00 × 10^6^ N·mm^2^ at 720 h and 2.04 ×10^6^ N·mm^2^ at 1000 h. Similar evolution was observed for secant stiffness, $K_{sec,R20}$, as evident from Table 12. Nevertheless, the 1000 h specimen remained mechanically stiffer than the unaged cable, with its apparent bending rigidity approximately 47% above the initial value. The evolution therefore indicates an initial aging-induced stiffening of the cable followed by partial loss of stiffness during prolonged thermal exposure. This initial increase in bending stiffness is in good agreement with the changes in dynamic and elastic stiffness independently observed by DMA and tensile testing respectively. The bending response at the specified minimum bending radius (geometrical approximation) provides further evidence of this behavior. The force required to reach the geometrically approximated 20 cm bending radius, $F_{R20}$, increased from 158.4 N in the unaged cable to 221.7 N after 240 h and reached 234.6 N after 500 h, corresponding to increases of approximately 40.0% and 48.1%, respectively, relative to the unaged state. The mechanical work required to reach the same bending condition, $U_{R20}$, increased even more strongly from 3.25 J initially to 6.13 J at 240 h and 6.55 J at 500 h, as shown in Table 12. Thus, after 500 h, the PFA cable required approximately twice the mechanical work of the unaged cable to be bent to the geometrically approximated minimum-radius condition. These changes are consistent with the increased modulus observed by DMA and the increasing rigidity and decreasing deformability observed in the tensile response. The maximum bending resistance exhibited a similar evolution.

$F_{peak}$ increased from 168.4 N at 0 h to 221.8 N after 240 h and 234.7 N after 500 h, followed by a decrease to 227.1 N at 720 h and 208.9 N after 1000 h. More importantly, thermal aging strongly affected the displacement at which the maximum bending resistance was reached. The unaged PFA cable reached its maximum force at 44.8 mm, well beyond the equivalent displacement, as highlighted in Figure 15a. In contrast, the 240, 500, and 720 h cables reached their peak forces at 35.4, 34.3, and 34.6 mm, respectively, i.e., approximately at the geometrically estimated reference displacement ($\delta_{R20}$) corresponding to the 20 cm minimum bending radius. This shift indicates that the aged PFA cable became progressively less compliant: substantially greater resistance was developed at lower bending deformation, but once this resistance was reached, further deformation produced little additional load-bearing capacity and, in several cases, a gradual reduction in force. The shift of $F_{peak}$ toward the geometrical equivalent minimum bending radius condition agrees well with the loss of ductility observed in tensile testing. Although thermal aging initially increased tensile resistance and bending stiffness, the accompanying reduction in elongation at break indicates progressively restricted capacity for large deformation. Thus, the increased bending force during the early-to-intermediate aging period should not be interpreted simply as an improvement in mechanical performance. Rather, the material becomes simultaneously stiffer and less deformable. This distinction is important for cable applications because high bending stiffness increases the force required during installation, whereas reduced elongation limits the ability of the polymer layers to accommodate local tensile and compressive strains generated during severe bending. Beyond $R_{20}$, several aged full-cable curves exhibit a plateau or decrease in force after $F_{peak}$, as highlighted in region C of Figure 15a. This post-peak load softening should not by itself be interpreted as direct evidence of fracture or irreversible material failure, because large-deformation cable bending can additionally involve geometrical reconfiguration, conductor-strand movement, interfacial slip, local cross-sectional distortion, and redistribution of load between the constituent layers [47,48,49]. Nevertheless, when considered together with the progressive reduction in tensile elongation and the macroscopic dimensional changes observed during thermal aging, the post-peak response indicates a reduction in the mechanical margin available for accommodating severe over-bending. This aspect can be discussed further together with the visual shrinkage and morphological observations when assessing the practical implications of prolonged thermal aging.

After approximately 500 h, the 3PB parameters began to decrease, as evident from Table 12. At 1000 h, ${EI}_{app}$, $K_{sec,R20}$, $F_{R20}$, and $U_{R20}$ had all declined from their intermediate-aging maxima, although they remained above their unaged values. This transition is also consistent with the longer-term behavior identified by the complementary thermal and mechanical analyses. At this stage, chain scission-assisted rearrangement or chemi-crystallization increasingly competes with simple physical annealing. This competing mechanism explains the apparently different characteristics observed after extended aging of PFA. Residual or chemi-crystallization-assisted ordering maintains the bending rigidity of the 1000 h PFA cable above the unaged value, whereas degradation-related loss of molecular connectivity progressively reduces the maximum rigidity achieved at 500 h. The same interpretation is supported by the DMA response, in which the initial increase in stiffness was followed by stabilization or reduction at prolonged aging, and by the tensile response, where the decrease in elongation became increasingly pronounced and the initial enhancement in tensile resistance was no longer sustained. Taken together, these observations suggest that the mechanical evolution cannot be represented by a simple monotonic degradation process. Instead, the measured behavior reflects competition between annealing/crystalline ordering, which increases stiffness, and chain scission-related degradation, which progressively reduces ductility and effective load-bearing continuity.

The jacket-removed specimens exhibited the same general non-monotonic aging tendency as the complete cable but showed substantially lower bending resistance. As shown in Table 12, the apparent bending rigidity of the jacket-removed configuration increased from 0.54 ×10^6^ N·mm^2^ in the unaged condition to 0.72 ×10^6^ N·mm^2^ at 240 h and reached 1.04 ×10^6^ N·mm^2^ at 500 h, before decreasing to 0.75 ×10^6^ N·mm^2^ and 0.67 ×10^6^ N·mm^2^ at 720 and 1000 h, respectively. Likewise, $F_{R20}$ increased from 77.0 N in the unaged condition to 103.2 N at 500 h before decreasing to 92.4 N at 1000 h, as illustrated in Figure 15b. The observation of the same aging sequence after jacket removal demonstrates that the conductor–primary-insulation assembly, including the primary PFA insulation, undergoes the annealing-induced stiffening and subsequent degradation-related mechanical evolution identified by DSC, DMA, and tensile testing. Nevertheless, comparison of the complete-cable and jacket-removed configurations demonstrates that the presence of the outer jacket and the associated structural configuration substantially increase the overall bending resistance. As summarized in Table 13, the difference in apparent bending rigidity between the two configurations, $\Delta EI_{config}$, was 0.85 ×10^6^ N·mm^2^ in the unaged condition, equivalent to 61.1% of the apparent rigidity of the complete cable. This difference increased to 2.42 ×10^6^ N·mm^2^ at 500 h, equivalent to 70% of the complete-cable value, and remained 1.37 ×10^6^ N·mm^2^ at 1000 h, corresponding to 67.1%. These differences should not be interpreted as the isolated mechanical contribution of the PFA jacket because jacket removal also changes the cable geometry, interfacial interactions, possible interlayer slip, and structural constraint. Rather, they quantify the change in the global bending response associated with the presence of the jacket and its integration within the multilayer cable structure. The relatively large difference in bending rigidity is nevertheless mechanically consistent with the outer radial position of the PFA jacket. Material located farther from the bending neutral axis can have a greater influence on the structural bending response. In the complete cable, however, this geometrical effect acts together with interfacial constraint and interaction among the cable layers; therefore, the individual effects cannot be separated from the present experiments. A similar behavior was observed at the geometrically approximated reference displacement $\delta_{R20}=34.6$ mm. In the unaged condition, $F_{R20}$ increased from 77.0 N for the jacket-removed configuration to 158.4 N for the complete cable, giving a difference of 81.4 N (see Table 13), equivalent to 51.3% of the complete-cable force. At 500 h, the corresponding difference increased to 131.4 N, equivalent to 56.0% of the complete-cable response, while at 1000 h it remained at 114.4 N, corresponding to 55.3%. Thus, the complete cable consistently required substantially greater force than the jacket-removed configuration at the same reference displacement. This difference reflects the combined influence of the outer jacket, altered cross-sectional geometry, interfacial constraint, and multilayer structural interaction rather than an independently measurable jacket force.

The bending work analysis further supports this distinction. $U_{R20}$ increased from 1.54 J in the jacket-removed configuration to 3.25 J in the complete cable in the unaged condition, from 2.09 to 6.55 J at 500 h, and from 1.79 to 4.75 J at 1000 h (Table 12). Consequently, the complete-cable configuration required approximately 111.0%, 213.3%, and 165.3% more work than the corresponding jacket-removed configuration at 0, 500, and 1000 h, respectively (Table 13). The largest difference occurred around the 500 h condition, broadly coinciding with the aging interval in which DSC and DMA indicated enhanced structural ordering and mechanical stiffening. However, these percentage differences represent configuration-level changes and cannot be assigned exclusively to the intrinsic mechanical behavior of the outer jacket.

A particularly important distinction between the two configurations is observed in the large-deformation region. In the jacket-removed specimens, $\delta_{peak}$ remained essentially constant at approximately 44.8–45.0 mm for all aging conditions, as evident from Figure 15b. By contrast, in the complete PFA cable, the peak shifted toward approximately 34–35 mm after 240–720 h, as shown in Figure 15a. The occurrence of this behavior only in the complete-cable configuration suggests that the outer jacket and the structural interactions introduced by its presence play an important role in governing the large-deflection response. Aging-induced changes in the stiffness and deformation capacity of the PFA jacket, together with changes in interlayer constraint and load transfer, may therefore contribute to the altered force–displacement response. Overall, the results demonstrate that the presence and aging state of the outer PFA jacket strongly influence the global bending behavior of the cable, although its independent mechanical contribution cannot be uniquely separated using the present jacket removal comparison.

Overall, the combined DSC, DMA, tensile and 3PB results therefore support a two-stage mechanical evolution of the PFA cable. During the first stage, extending approximately through the intermediate aging period, thermal annealing promotes chain rearrangement and crystalline ordering. Reduced amorphous-chain mobility produces higher DMA stiffness, increased tensile resistance, substantially increased apparent bending rigidity, and greater force and energy requirements during cable bending. During prolonged aging, degradation becomes increasingly important. Chain scission can facilitate further local ordering or chemi-crystallization but simultaneously reduces molecular connectivity, tie-chain effectiveness, and ductility. As a result, the PFA cable remains more crystalline and structurally stiffer than in the unaged state, yet its bending rigidity decreases from the intermediate-aging maximum and its ability to accommodate large deformation continues to deteriorate.

## 4. Discussion

### 4.1. Aging Mechanism and Evolution of PFA Performance

**Figure 16:**
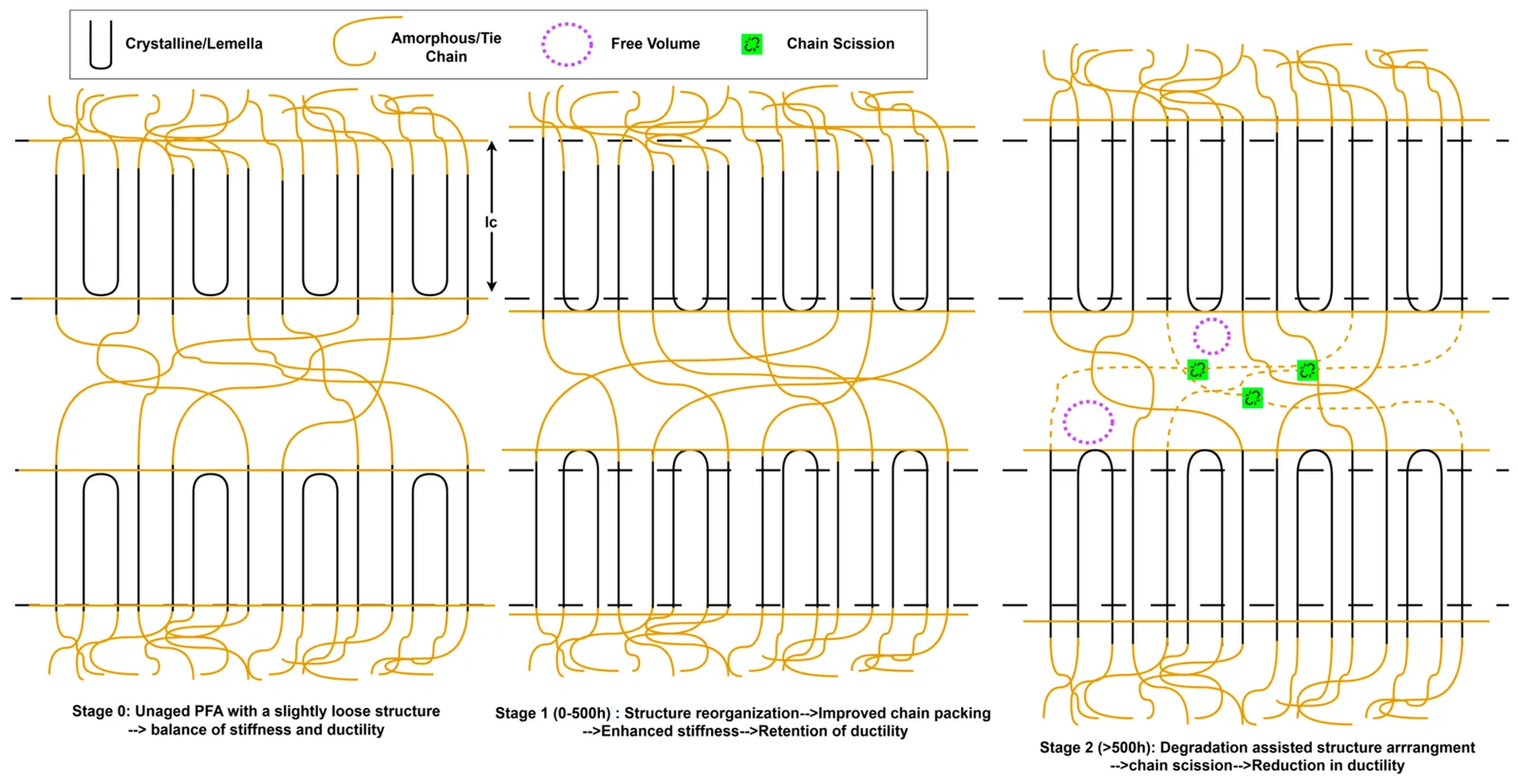
Evolution of possible structural changes in PFA at different stages of aging process.

The combined thermal, mechanical, morphological, and macroscopic results indicate that thermal aging of the investigated PFA cable does not proceed through a single monotonic degradation process. Instead, the evolution can be interpreted using the two-stage mechanism schematically illustrated in Figure 16. The combined aging mechanism proposed in Figure 16 agrees well with the thermal, mechanical, and morphological results obtained in this study. During the first stage (0–500 h), thermal exposure primarily promotes annealing-induced structural reorganization, including improved chain packing and crystalline development (increased lamellar thickness $I_{c}$), while substantial molecular connectivity is retained, as shown in Figure 16. This interpretation is in agreement with the increase in crystallinity observed by DSC and the corresponding increases in storage modulus, tensile modulus, and stiffness obtained from DMA and tensile testing. At longer nominal elapsed durations (720–1000 h), the contribution of chain scission becomes increasingly important, particularly within the amorphous regions, as illustrated in Figure 16. The resulting shorter chain segments can undergo further rearrangement and crystallization, producing a degradation-assisted or chemi-crystallization stage. These observations suggest that degradation-related processes increasingly compete with structural ordering at prolonged exposure. Consequently, the PFA becomes progressively stiffer while its ability to accommodate deformation decreases, indicating that increased crystallinity and modulus at prolonged aging times should not necessarily be interpreted as improved mechanical durability.

**Figure 17:**
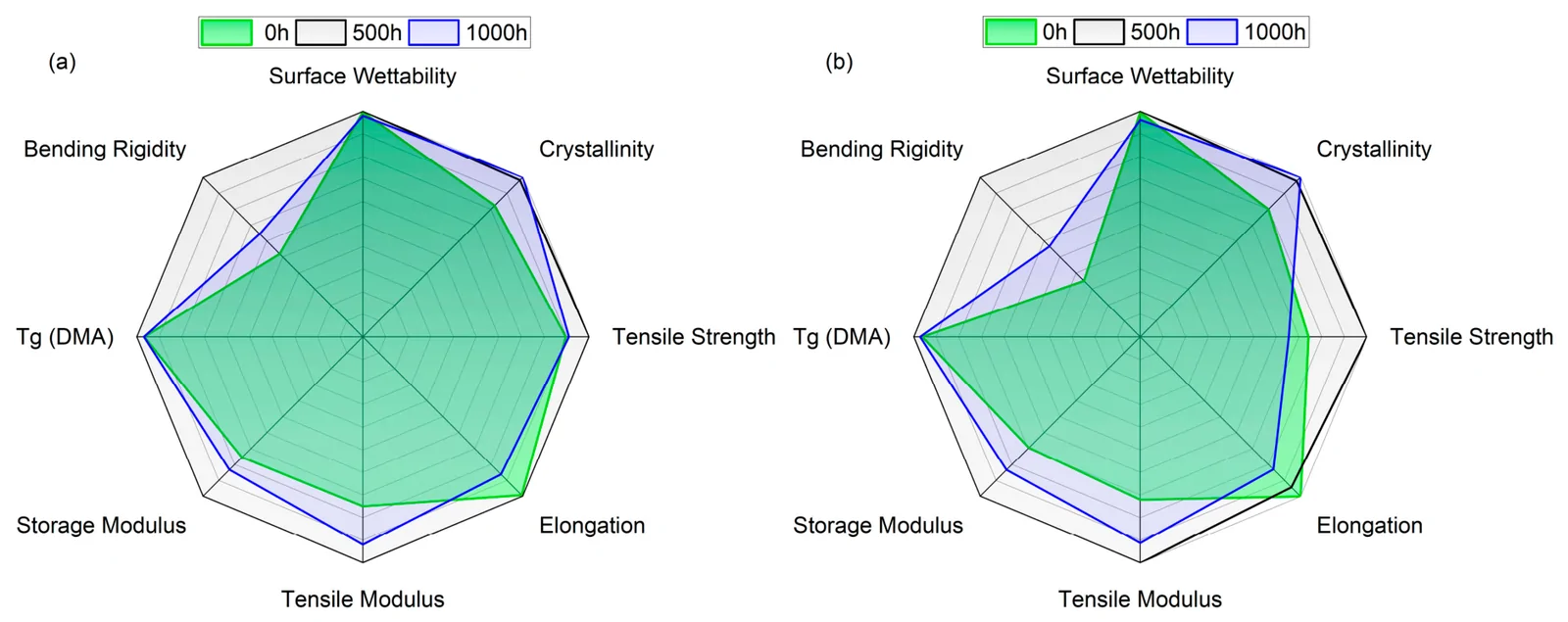
Normalized performance radar charts of PFA; each parameter was normalized by its maximum value across all aging conditions, yielding a dimensionless scale for direct comparison among parameters with different units. (**a**) Performance radar chart of PFA insulation at different aging times. (**b**) Performance radar chart of PFA jacket.

This transition is further summarized by the normalized performance maps for the PFA insulation and jacket in Figure 17a and Figure 17b, respectively. For the insulation (Figure 17a), aging is characterized mainly by increased crystallinity, tensile modulus, storage modulus, and bending rigidity, while elongation remains comparatively well retained. The PFA jacket (Figure 17b) showed a more pronounced evolution of crystallinity and stiffness-related properties together with a greater reduction in elongation, indicating that it undergoes stronger structural modification than the primary insulation. The comparatively limited changes in surface wettability and DMA transition temperature suggest that the most pronounced changes occur through bulk structural rearrangement and crystalline organization, rather than through extensive alteration of the basic PFA chemical structure. These trends are consistent with the DSC, XRD, TG-MS, SEM, DMA, tensile, and bending results discussed previously and demonstrate that the aged cable evolves from an initially ductile semicrystalline structure toward a more highly ordered and mechanically rigid state with progressively reduced deformation capability. The comparatively stronger evolution of the PFA jacket also explains its significant contribution to the overall flexural stiffness of the complete cable.

### 4.2. Implications for Cable Design and Mechanical Handling

The axial shrink-back observed during visual inspection, discussed in Section 3.1, has direct implications for cable termination design. Both straight and bent PFA cable specimens exhibited progressive insulation retraction, with a greater dimensional change occurring in the outer PFA jacket and a total shrink-back approaching approximately 10 mm after a nominal elapsed duration of 1000 h. Although such severe thermal exposure is considerably greater than normal aircraft operating conditions, shrinkage remains relevant because practical cable runs may extend over several meters and contain bends, clamps, vibration, and local thermal gradients. These combined effects may redistribute axial stresses and promote local movement of the insulation relative to the conductor. Therefore, termination designs should provide sufficient axial restraint and strain-relief allowance to prevent insulation withdrawal, conductor exposure, or alteration of the designed termination geometry during long-term service.

The 3PB response in Figure 15a further demonstrates that thermal aging increases the initial flexural rigidity of the PFA cable, consistent with the increased crystallinity and modulus identified in their respective analyses. However, when bending progresses beyond the recommended bending radius, the force–displacement response enters a nonlinear region followed by load softening or a near-force plateau, as discussed previously in 3PB analysis. This behavior indicates that further deformation is no longer accommodated predominantly by elastic bending and may instead involve local structural rearrangement or irreversible deformation. Since prolonged aging simultaneously increases stiffness and reduces ductility, aged PFA cables may become more susceptible to localized mechanical damage during excessive bending. Therefore, an appropriate safety margin above the specified minimum bending radius should be maintained during installation, while additional care is required when removing, repositioning, or replacing thermally aged PFA cables. Such a margin is particularly important in aircraft applications, where vibration and cyclic movement may locally increase cable curvature during operation.

### 4.3. Mechanical End-of-Life and Relevance to Service Conditions

Despite the structural and mechanical changes observed in the relevant analyses, neither the PFA insulation nor the jacket reached the selected mechanical end-of-life criterion after a nominal elapsed duration of 1000 h. The elongation retention remained approximately 86.9% for the PFA insulation and 83.03% for the jacket, both above the conventional 50% criterion and the more conservative 70% retention level considered in previous studies [5,50]. This indicates that degradation had initiated but had not progressed to mechanical end-of-life under the investigated condition. The distinction between accelerated aging time and actual service time is important in interpreting the present results. As demonstrated in the referenced XLPE study, 4.7 years of field operation was estimated to correspond to only 16.9 h of accelerated laboratory aging, illustrating the large difference between laboratory aging duration and real service exposure under lower operating stresses [51]. Although this relationship cannot be directly transferred to PFA, the same principle applies to thermal aging, where the exposure at 280 °C used in the present study is substantially more severe than the expected continuous operating temperature of approximately 200 °C and short-duration excursions toward 240 °C [5,7]. However, quantitative conversion of the accelerated aging duration to actual PFA cable service life therefore requires experimentally determined activation energy and Arrhenius-based time–temperature extrapolation. Therefore, the present results demonstrate that the mechanical end-of-life of the PFA-insulated cable was not reached within a nominal elapsed duration of 1000 h.

## 5. Conclusions

This study demonstrates that thermal aging of a complete PFA-insulated cable involves coupled material- and cable-level changes that cannot be fully captured using isolated polymer specimens. Over the investigated aging intervals, the earlier 0–500 h response was dominated by annealing and structural ordering, whereas degradation-related effects became increasingly evident at the later 720–1000 h conditions. At the cable scale, axial shrink-back was the most prominent dimensional change, while the bending response showed non-monotonic variations in force, secant stiffness, bending work, and apparent structural bending rigidity. The outer PFA jacket exhibited more pronounced aging-related changes than the primary insulation and substantially influenced the overall bending response of the multilayer cable. Nevertheless, the fluorinated backbone remained largely preserved, extensive chemical degradation was not observed, and the mechanical end-of-life criterion was not reached within the investigated aging period.

The principal contribution of this work is the correlation of aging in the individual PFA layers with the dimensional and mechanical response of the complete conductor–insulation–jacket assembly. The results identify axial shrink-back, layer-dependent aging, and changes in cable bending behavior as useful indicators for durability assessment of high-temperature aerospace cables. From a design perspective, these findings highlight the importance of accommodating axial dimensional change, controlling bending conditions, accounting for different aging sensitivities of the insulation and jacket, and ensuring adequate thermal management. The present study was limited to cyclic aging at a single maximum temperature; therefore, quantitative service life prediction will require longer exposures, multiple aging temperatures, and ultimately combined thermal, electrical, mechanical, pressure, humidity, and vibration stresses representative of aerospace operation.

## Figures and Tables

**Table 1:** Cyclic thermal aging conditions, completed cycles, elapsed aging time, and cumulative high-temperature exposure of PFA cable specimens.

Nominal Condition	Completed Cycles	Actual Elapsed Time	8 h Heating/High-Temp Segment	Approx. 280 °C Dwell
0 h	0	0 h	0 h	0 h
240 h	10	240 h	80 h	≈70 h
500 h	21	504 h	168 h	≈147 h
720 h	30	720 h	240 h	≈210 h
1000 h	42	1008 h	336 h	≈294 h

**Table 2:** Visual observations and dimensional changes in straight and bent PFA-insulated cables during thermal aging.

	Parameter	Unaged	240 h	500 h	720 h	1000 h
Straight cable	Initial specimen length, $L_{0}$ (mm)	300	300	300	300	300
	End retraction—straight cable (mm/end)	0	3	4	4–5	≈5
	Estimated axial shrink-back—straight cable (%)	0	2.00	2.67	2.67–3.33	≈3.33
Bent cable	Cable length present at inspection (m)	5.00	5.00	3.75	2.50	1.25
	Local terminal retraction (mm/end) ^1^	0	5–6	7–8	9–10	≈10
General visual observations	Outer diameter change	-	Not measurable	Not measurable	Not measurable	Not measurable
	PFA surface appearance	Smooth and glossy	No visible change	No visible change	Slight dulling	Slight dulling
	PFA color change	-	No visible change	No visible change	No visible change	No visible change
	Macroscopic surface cracking	None	Not observed	Not observed	Not observed	Not observed
	Visible radial delamination	None	Not observed	Not observed	Not observed	Not observed
	Mass change	-	Not measurable	Not measurable	Not measurable	Not measurable
	Overall visual condition	Reference condition	End retraction initiated	Progressive retraction	Retraction approaching stabilization	Retraction approximately stabilized

^1^ The bent cable values represent local terminal retraction only. They were not normalized or quantitatively compared with the straight cable shrinkage because the bent specimen was sequentially sectioned and did not retain a constant gauge length.

**Table 3:** DSC melting and crystallization parameters of PFA insulation at different aging times.

Aging Time (h)	T_onset_ (°C)	T_m_ (°C)	I_c_ (nm)	ΔH_m_ (J/g)	X_c_ (%)	T_c_ (°C)
0	301.6	309.2	5.70	30.4	45.3	286.0
240	303.6	311.8	6.29	36.8	54.9	286.3
500	304.2	312.1	6.37	37.8	56.4	286.7
720	304.7	312.6	6.50	38.1	56.9	286.9
1000	302.0	313.0	6.61	38.3	57.1	287.2

**Table 4:** Comparison of crystallinity evolution of PFA insulation and jacket at different aging times.

Aging Time (h)	PFA Insulation		PFA Jacket	
	ΔH_m_	X_c_ (%)	ΔH_m_	X_c_ (%)
0	30.8	46.0	30.5	45.5
500	36.7	54.8	37.2	55.5
1000	37.5	55.9	38.1	56.9

**Table 5:** XRD-derived structural parameters of PFA insulation as a function of thermal aging time.

Aging Time (h)	$2\theta_{100}$	$d_{100}$ (Å)	$2\theta_{110}$	$2\theta_{200}$	$D_{100}$ (nm)
0	17.5	5.064	30.9	35.9	13.86
240	17.6	5.035	31.1	36.2	14.89
500	17.7	5.007	31.1	36.3	16.08
720	17.7	5.007	31.2	36.3	15.46
1000	17.7	5.007	31.2	36.3	10.18

**Table 6:** TGA and DTG parameters of PFA insulation and jacket at different aging times.

Aging Time (h)	T_5%_ (°C)	T_30%_ (°C)	T_max_ (°C)	THRI (°C)
0	529.0	554.0	574.9	266.5
240	528.4	553.5	574.5	266.3
500	528.3	553.4	574.2	266.2
720	526.3	552.5	572.8	265.6
1000	525.8	550.7	570.4	264.9
0-PFA-Jacket	527.5	553.7	573.5	266.1
1000-PFA-Jacket	521.9	548.5	568.5	263.6

**Table 7:** TG–MS parameters of characteristic evolved-gas fragments from unaged and thermally aged PFA insulation.

Fragment	Probable Parent Molecule(s)	0 h			1000 h		
		Peak Height (×10^−12^ A mg^−1^)	Peak Temp. (°C)	Peak Area (×10^−11^ A °C mg^−1^)	Peak Height (×10^−12^ A mg^−1^)	Peak Temp. (°C)	Peak Area (×10^−11^ A °C mg^−1^)
CF2+	CF/CF_2_-related	0.48	567	1.50	0.53	559	1.80
HF+	HF	1.11	571	4.29	1.64	563	6.14
COF2+	COF_2_	0.74	563	2.31	1.57	559	5.55
CFO+	COF/COF_2_-related	7.23	563	23.8	11.7	559	44.0
CF3+	CF_3_/CF_2_-related	0.95	563	3.25	1.10	563	4.13

**Table 8:** EDS surface scan elemental analysis of PFA insulation at different aging times.

Samples	Weight (%)			Atomic (%)		
	0 h	500 h	1000 h	0 h	500 h	1000 h
C	19.1	19.44	20.4	27.15	27.42	28.27
O	0.50	0.54	0.60	0.65	0.68	0.73
F	80.4	80.02	79.0	72.2	71.9	71.0

**Table 9:** EDS surface scan elemental analysis of PFA jacket at different aging times.

Samples	Weight (%)		Atomic (%)	
	0 h	1000 h	0 h	1000 h
C	16.12	17.8	23.28	24.8
O	0.64	0.72	0.69	0.75
F	83.24	81.48	76.03	74.45

**Table 10:** DMA test parameters of PFA insulation and jacket at different aging times.

Aging Time (h)	PFA Insulation		PFA Jacket	
	Storage Modulus at T_g_ (MPa)	T_g_ Peak (°C)	Storage Modulus at T_g_ (MPa)	T_g_ Peak (°C)
0	208.7	86.5	208.3	86.5
240	256.0	87.7	269.5	88.6
500	276.7	89.6	300.1	90.0
720	264.0	88.2	291.6	89.1
1000	230.5	86.6	250.2	87.3

**Table 11:** Tensile test parameters of PFA insulation and jacket at different aging times.

Aging Time (h)	PFA Insulation				PFA Jacket			
	Tensile Strength (MPa)	Young Modulus (MPa)	Yield Stress (MPa)	Elongation (%)	Tensile Strength (MPa)	Young Modulus (MPa)	Yield Stress (MPa)	Elongation (%)
0	12.56 ± 1.50	252	9.5	319.9 ± 19	11.76 ± 1.00	249	10.0	324.1 ± 23
240	13.53 ± 1.20	325	10.5	321.1 ± 22	15.73 ± 1.50	331	10.6	315.0 ± 20
500	14.02 ± 1.00	336	10.7	322.7 ± 24	15.85 ± 1.20	345	12.5	305.5 ± 25
720	13.31 ± 1.30	328	9.8	314.8 ± 21	13.30 ± 1.70	335	10.1	275.2 ± 27
1000	12.75 ± 1.60	309	9.1	278.0 ± 26	10.38 ± 1.40	315	9.9	269.1 ± 24

**Table 12:** 3PB test results of PFA cable configurations at different aging times.

Aging Time (h)	Configuration	$F_{R20}$ (N)	$F_{peak}$ (N)	$\delta_{peak}$ (mm)	$EI_{app}$ (×10^6^.N.mm^2^)	$K_{sec,R20}$ (N/mm)	$U_{R20}$ (J)
0	Full cable	158.4	168.4	44.8	1.39	4.58	3.25
	Jacket removed	77.0	83.0	45.0	0.54	2.23	1.54
240	Full cable	221.7	221.8	35.4	2.96	6.41	6.13
	Jacket removed	95.6	110.1	45.0	0.72	2.76	1.78
500	Full cable	234.6	234.7	34.3	3.46	6.78	6.55
	Jacket removed	103.2	112.5	44.8	1.04	2.98	2.09
720	Full cable	227.0	227.1	34.6	3.00	6.56	6.31
	Jacket removed	100.1	111.6	44.9	0.75	2.89	1.95
1000	Full cable	206.8	208.9	39.1	2.04	5.98	4.75
	Jacket removed	92.4	106.6	45.0	0.67	2.67	1.79

**Table 13:** Differences in bending response between complete-cable and jacket-removed configurations.

Aging Time (h)	$\Delta EI_{config}$ (×10^6^.N.mm^2^)	Difference Relative to Full Cable $EI_{app}$ (%)	$\Delta F_{R20}$ (N)	Difference Relative to Full Cable $F_{R20}$ (%)	$\Delta U_{R20}$ (J)	Increase in $U_{R20}$ (%)
0	0.85	61.1	81.4	51.3	1.71	111.0
500	2.42	69.9	131.4	56.0	4.46	213.3
1000	1.37	67.1	114.4	55.3	2.96	165.3

## Data Availability

The original contributions presented in this study are included in the article. Further inquiries can be directed to the corresponding authors.

## References

[1] Borghei M., Ghassemi M. (2021). Insulation materials and systems for more-and all-electric aircraft: A review identifying challenges and future research needs. IEEE Trans. Transp. Electrif..

[2] Lebey T., Rumi A., Cavallini A. (2022). Challenges for electrical insulation systems in high voltage aviation applications. IEEE Electr. Insul. Mag..

[3] Zhang X.-M., Liu J.-G., Yang S.-Y. (2016). A review on recent progress of R&D for high-temperature resistant polymer dielectrics and their applications in electrical and electronic insulation. Rev. Adv. Mater. Sci..

[4] Henri V., Chedaille J., Henrard P., Dantras E. (2026). Long-Time Thermal Aging of Perfluoroalkoxy Polymers for Electrical Insulation: Structural and Physical Properties Evolutions. J. Appl. Polym. Sci..

[5] Ahmad J., Niasar M.G. (2026). Comprehensive investigation of thermal aging mechanisms in PFA for high-voltage cable insulation in hybrid-electric aircraft. Polym. Test..

[6] Guo R., Dong J., Wolleswinkel R.E., de Vries R., Niasar M.G. (2024). Electrical architecture of 90-seater electric aircraft: A cable perspective. IEEE Trans. Transp. Electrif..

[7] Xu T., Ahmad J., Liang Y., Mouli G.R.C., Niasar M.G. (2026). Design of Electrical Power Architectures for All-Electric Aircraft with Emphasis on Cabling and Battery Systems. IEEE Trans. Transp. Electrif..

[8] Ahmad J., Niasar M.G. (2025). Aging behavior of PEEK, PTFE, and PI insulation materials under thermal oxidative and humid conditions for aerospace applications. J. Appl. Polym. Sci..

[9] Jiang J., Li Z., Li W., Ranjan P., Wei X., Zhang X., Zhang C. (2023). A review on insulation challenges towards electrification of aircraft. High Volt..

[10] Chikhoune Y., Teyssedre G., Berquez L., Collin P. (2024). Thermal Ageing of an Extrudable Fluorinated Polymer for Aerospace Application. 2024 IEEE 5th International Conference on Dielectrics (ICD).

[11] Deng H., Bao C., Zhang X., An K., Xiao C., Zheng K. (2026). Annealing Temperature Effects on the Perfluoroalkoxy Lamellar Thickness and Mechanical Properties. J. Polym. Sci..

[12] Endo M., Yamada K., Tadano K., Nishino Y., Yano S. (2000). Miscibility and crystallization of polytetrafluoro-ethylene/poly(tetrafluoroethylene-co-perfluoropropylvinyl ether) blends. Macromol. Rapid Commun..

[13] Gao X., Pan Z., Fu C., Liu S., Pan Y., Zhang R. (2026). Synergistic Enhancement of Electrical, Thermal, and Mechanical Properties in PFA Composites via Graphene/Multi-Walled Carbon Nanotube Hybrid Fillers. Polym. Eng. Sci..

[14] Chemours (2024). Teflon™ PFA Fluoropolymer Film—Properties Bulletin.

[15] Neveux B., Rivenc J., Castellon J., Agnel S., Laurentie J.-C., Forget F., Doumerg R., Pommier L., Peres G. (2026). Impact of a Thermo-Electric Ageing Under Low Pressure Environment on Space Charges in Fluoropolymer Insulation for HVDC Aeronautical Cables. 2026 IEEE 6th International Conference on Dielectrics (ICD).

[16] Legeay G., Coudreuse A., Legeais J.-M., Werner L., Bulou A., Buzaré J.-Y., Emery J., Silly G. (1998). AF fluoropolymer for optical use: Spectroscopic and surface energystudies; comparison with other fluoropolymers. Eur. Polym. J..

[17] Monson L., Moon S.I., Extrand C.W. (2009). Gas permeation resistance of various grades of perfluoroalkoxy–polytetrafluoroethylene copolymers. J. Appl. Polym. Sci..

[18] Perelygin I.S., Peskova M.Z., Zakirov Z.Z., Glinkin I.M. (1976). Determination of the crystallinity of polytetrafluoroethylene from infrared absorption spectra. J. Appl. Spectrosc..

[19] Huang X., Martinez-Vega J., Malec D. (2013). Morphological evolution of polytetrafluoroethylene (PTFE) during thermal-oxidative ageing above and below the melting temperature. 2013 IEEE International Conference on Solid Dielectrics (ICSD).

[20] Peacock C.J., Hendra P.J., Willis H.A., Cudby M.E.A. (1970). Raman spectrum and vibrational assignment for poly(tetrafluoroethylene). J. Chem. Soc. A Inorg. Phys. Theor..

[21] Bracciale M.P., Capasso L., Sarasini F., Tirillò J., Santarelli M.L. (2022). Effect of aging on the mechanical properties of highly transparent fluoropolymers for the conservation of archaeological sites. Polymers.

[22] Mihály J., Sterkel S., Ortner H.M., Kocsis L., Hajba L., Furdyga É., Mink J. (2006). FTIR and FT-Raman spectroscopic study on polymer based high pressure digestion vessels. Croat. Chem. Acta.

[23] Strabelli P.G., Sciuti V.F., Montilha F.S., Canto L.B., Canto R.B. (2014). Effects of sintering variables on the microstructure of isostatically pressed PTFE parts. Polímeros.

[24] Romoaldo C.H., Francisquetti E.L., Simon D.A., Ernze J.R., Bischoff E. (2023). Recycling tetrafluoroethylene–Perfluoroalkyl vinylether copolymer (PFA) using extrusion process. Macromol. Mater. Eng..

[25] Huang X.L., Martinez-Vega J., Malec D. (2014). Morphological evolution of polytetrafluoroethylene in extreme temperature conditions for aerospace applications. J. Appl. Polym. Sci..

[26] Aal M.M.A.E., Mokhtar S.M., Osman M.B.S. (1996). Dielectric relaxation of poly(tetrafluoroethylene–perfluorovinyl ether) films. Polym. Int..

[27] Fujimori A., Hasegawa M., Masuko T. (2007). Spherulitic structures of poly [tetrafluoroethylene-co-(perfluoroalkyl vinyl ether)]. Polym. Int..

[28] Endo M., Ohnishi A., Kutsumizu S., Shimizu T., Yano S. (2004). Crystallization in binary blends of polytetrafluoroethylene with perfluorinated tetrafluoroethylene copolymer. Polym. J..

[29] Katoueizaheh E., Rajabinejad H., Rafferty A., Morris M.A. (2025). Impact of sintering temperature and compression load on the crystallinity and structural ordering of polytetrafluoroethylene. RSC Adv..

[30] Gu J., Li N., Tian L., Lv Z., Zhang Q. (2015). High thermal conductivity graphite nanoplatelet/UHMWPE nanocomposites. RSC Adv..

[31] Parada M.A., Minamisawa R.A., Moreira M.V., de Almeida A., Muntele I., Ila D. (2007). Damage effects of gamma and X-rays in polymer film electrets. Surf. Coat. Technol..

[32] Hossain U.H., Muench F., Ensinger W. (2014). A comparative study on degradation characteristics of fluoropolymers irradiated by high energy heavy ions. RSC Adv..

[33] Lappan U., Geißler U., Scheler U., Lunkwitz K. (2003). Identification of new chemical structures in poly(tetrafluoroethylene-co-perfluoropropyl vinyl ether) irradiated in vacuum at different temperatures. Radiat. Phys. Chem..

[34] Gehrmann H., Nuredin V., Aleksandrov K., Tonndorf S., Hauser M., Meermann B., Stapf D. (2026). Thermal Degradation of PFAS in Different Scales: Measurement of Hydrogen Fluoride (HF). Chem. Ing. Tech..

[35] Jin J., Fabiani D., Suraci S.V. (2026). High temperature performance of polyimide under accelerated thermal aging: Linking chemical modifications to electrical properties. Polym. Degrad. Stab..

[36] Bouguedad D., Mouri D., Hadjadj A. (2026). Quantitative Kinetic Analysis of Hydraulic Aging in EPDM Rubber: Evolution of Functional Properties. Polymers.

[37] Wenzel R.N. (1936). Resistance of solid surfaces to wetting by water. Ind. Eng. Chem..

[38] Vesel A., Zaplotnik R., Primc G., Mozetič M. (2024). Kinetics of surface wettability of aromatic polymers (PET, PS, PEEK, and PPS) upon treatment with neutral oxygen atoms from non-equilibrium oxygen plasma. Polymers.

[39] Lopez G. (2021). High-performance polymers for aeronautic wires insulation: Current uses and future prospects. Recent Prog. Mater..

[40] Yin C., Miranda D.F., Zhang S., Zhang Q., Runt J. (2016). Microstructure evolution of poly[tetrafluoroethylene-co-(perfluoropropylvinylether)] films under uniaxial deformation. Polymer.

[41] Kuzak S.G., Shanmugam A. (1999). Dynamic mechanical analysis of fiber-reinforced phenolics. J. Appl. Polym. Sci..

[42] Huang X., Martinez-Vega J., Malec D. (2013). Dielectric breakdown and morphological evolution of PTFE during thermal-oxidative ageing at temperatures lower and higher than the melting temperature. 2013 Annual Report Conference on Electrical Insulation and Dielectric Phenomena.

[43] Vilaplana F., Karlsson S. (2008). Quality concepts for the improved use of recycled polymeric materials: A review. Macromol. Mater. Eng..

[44] Song F., Wang Q., Wang T. (2016). The effects of crystallinity on the mechanical properties and the limiting PV (pressure × velocity) value of PTFE. Tribol. Int..

[45] Fayolle B., Richaud E., Colin X., Verdu J. (2008). Degradation-induced embrittlement in semi-crystalline polymers having their amorphous phase in rubbery state. J. Mater. Sci..

[46] (1997). Standard Test Methods for Flexural Properties of Unreinforced and Reinforced Plastics and Electrical Insulating Materials.

[47] Li H., Wang Z., Peng Y., Wang Y., Guo Z., Ringsberg J.W. (2027). A Theoretical Model for the Thermo-Mechanical Bending Behavior of Submarine Power Cables. Mar. Struct..

[48] Fang P., Li X., Jiang X., Hopman H., Bai Y. (2026). Thermo-electro-mechanical bending of submarine power cables. Eng. Struct..

[49] Qiao D., Zou G., Chen H., Tang G., Lu L., Ou J. (2025). Influence of thermo-mechanical coupling effects on the stiffness characteristics of dynamic cables. Appl. Ocean Res..

[50] Liu Z., Hao J., Liao R., Li J., Gao Z., Liang Z. (2019). Morphological, structural, and dielectric properties of thermally aged AC 500 kV XLPE submarine cable insulation material and its deterioration condition assessment. IEEE Access.

[51] Liu Y., Yu J., Ma M. (2026). Performance Evolution and Ageing Characteristics of Crosslinked Polyethylene Insulation for HVDC Cables After Field Operation. High Volt..

